# RNA polymerase mutations that facilitate replication progression in the *rep uvrD recF* mutant lacking two accessory replicative helicases

**DOI:** 10.1111/j.1365-2958.2010.07208.x

**Published:** 2010-07

**Authors:** Zeynep Baharoglu, Roxane Lestini, Stéphane Duigou, Bénédicte Michel

**Affiliations:** 1 CNRS, Centre de Génétique MoléculaireFRE 3144, Gif-sur-Yvette F-91198, France; 2 Université Paris-SudOrsay F-91405, France

## Abstract

We observed that cells lacking Rep and UvrD, two replication accessory helicases, and the recombination protein RecF are cryo-sensitive on rich medium. We isolated five mutations that suppress this Luria–Bertani (LB)-cryo-sensitivity and show that they map in the genes encoding the RNA polymerase subunits RpoB and RpoC. These *rpoB* (D444G, H447R and N518D) and *rpoC* mutants (H113R and P451L) were characterized. *rpoB*^H447R^ and *rpoB*^D444G^ prevent activation of the P*rrn* core promoter in rich medium, but only *rpoB*^H447R^ also suppresses the auxotrophy of a *relA spoT* mutant (stringent-like phenotype). *rpoC*^H113R^ suppresses the thermo-sensitivity of a *greA greB* mutant, suggesting that it destabilizes stalled elongation complexes. All mutations but *rpoC*^P451L^ prevent R-loop formation. We propose that these *rpo* mutations allow replication in the absence of Rep and UvrD by destabilizing RNA Pol upon replication–transcription collisions. In a RecF^+^ context, they improve growth of *rep uvrD* cells only if DinG is present, supporting the hypothesis that Rep, UvrD and DinG facilitate progression of the replication fork across transcribed sequences. They rescue *rep uvrD dinG recF* cells, indicating that in a *recF* mutant replication forks arrested by unstable transcription complexes can restart without any of the three known replication accessory helicases Rep, UvrD and DinG.

## Introduction

Replication forks are susceptible to be arrested by a variety of obstacles, including DNA-bound proteins such as RNA polymerases (RNA Pol) (Mirkin and Mirkin, 2007; Rudolph *et al*., 2007). DNA instability is associated with replication fork arrest (reviewed in Aguilera and Gomez-Gonzalez, 2008), and in order to limit the deleterious consequences of replication–transcription collisions, cells encode enzymes that facilitate replication through transcription units. In yeast, the Rrm3 helicase travels with the replication fork machinery and dislodges RNA Pols from tRNA and rRNA genes as well as tightly bound proteins from heterochromatin (Azvolinsky *et al*., 2009 and references therein). In *Escherichia coli*, this function was originally ascribed to the Rep helicase; first, based on the observations that in the *rep* mutant chromosome replication is slowed down and requires specific functions (Lane and Denhardt, 1975; Seigneur *et al*., 1998; Petit and Ehrlich, 2002), and second, because the purified Rep helicase is specifically capable of dislodging DNA-bound proteins (Yancey-Wrona and Matson, 1992). More recently, evidence was provided that transcribed sequences are indeed a major obstacle to replication in rapidly growing cells, and that Rep is the main, although not the only, helicase that assists replication progression across highly transcribed sequences (Guy *et al*., 2009; Boubakri *et al*., 2010).

Rep and UvrD are two 3′ to 5′ helicases that share 40% homology. The existence of a redundant function for these paralogues has been suspected since the original observation that the *rep uvrD* double mutant shows severe growth defects (Taucher-Scholtz *et al*., 1983). However, the situation turned out to be more complex and UvrD is now known to play two different roles in *rep* mutants, revealed by the two classes of mutations that suppress the growth defects of the *rep uvrD* double mutant. The first class of mutations that was identified inactivates the RecFOR recombination pathway (Petit and Ehrlich, 2002). Combined with the observation that UvrD can remove RecA from ssDNA *in vitro*, the rescue of *rep uvrD* cells by the inactivation of recombination proteins led to the proposal that UvrD is essential in *rep* mutants because it removes deleterious RecFOR-dependent RecA-filaments that assemble at blocked replication forks (Veaute *et al*., 2005; Lestini and Michel, 2008). However, the nature of the obstacles arresting replication in the first place, thus allowing RecFOR-RecA binding to forks in *rep* mutants, remained unknown. Moreover, the suppression of *rep uvrD* co-lethality by *recFOR* inactivation was shown to be only partial, confirming the existence of an obstacle to replication restart other than RecFOR-RecA bound to DNA in *rep uvrD* cells (Guy *et al*., 2009; Boubakri *et al*., 2010). Two lines of evidence identified RNA polymerases as the original cause of replication arrest in *rep uvrD* cells: first, mutations that map in the RNA Pol genes *rpoB* and *rpoC* were shown to suppress the growth defects of this double mutant (Guy *et al*., 2009; Boubakri *et al*., 2010); second, replication arrest sites were directly visualized in *rep* and *rep uvrD recF* mutants at inverted ribosomal operons (*rrn*), provided that these operons were facing replication and were highly transcribed (Boubakri *et al*., 2010). Rep was confirmed to be the major accessory helicase and was shown to be attracted to replication forks by a direct interaction with the replicative helicase DnaB (Guy *et al*., 2009). In addition to UvrD, a third player was identified, the 5′ to 3′ helicase DinG (Boubakri *et al*., 2010). DinG is essential for the viability of the *rep uvrD recF* mutant and it was proposed that, provided that *recF* is inactivated, DinG can clear RNA Pols from blocked replication forks in the absence of both Rep and UvrD. In addition, DinG is essential for the viability of *rep* and *uvrD* single mutants when replication collides with RNA Pol at highly expressed inverted *rrn*, indicating that the presence of two of these three helicases is required when replication–transcription collisions are increased. Finally, DinG has the specific function of unwinding RNA–DNA hybrids *in vivo,* as it is also essential for viability when replication forks are arrested by R-loops (Boubakri *et al*., 2010).

The described RNA Pol mutations that suppress *rep uvrD* growth defects are mutations that constitutively confer phenotypes akin to the induction of the stringent response (Guy *et al*., 2009; Boubakri *et al*., 2010). The stringent response is an adaptation to amino acid starvation through the induction of the alarmone ppGpp (reviewed in Potrykus and Cashel, 2008). Binding of ppGpp to RNA Pol, as well as mutations that mimic this binding, affect transcription initiation from specific promoters, which decreases the expression of ribosomal operons (*rrn*) and activates the expression of amino acids biosynthetic genes in *relA spoT* double mutants, allowing their growth in minimal medium without amino acids (Bartlett *et al*., 1998; 2000; Zhou and Jin, 1998; Barker *et al*., 2001a,b;). The so-called ‘stringent mutations’ in RNA Pol mimic the presence of ppGpp in decreasing the half-life of open complexes. Although we have not measured the half-life of transcription open complexes, we will call here ‘stringent-like’ the phenotype conferred by RNA Pol mutations that both decrease *rrn* expression in rich medium and allow growth of *relA spoT* double mutants on minimal medium. RNA Pol mutations that increase transcription of amino acid biosynthetic promoters also destabilize transcription elongation complexes (TEC) (Trautinger and Lloyd, 2002; Trautinger *et al*., 2005). Such mutations were thought to suppress the lethality of *rep uvrD* double mutants owing to the destabilization of RNA Pol–DNA complexes (Guy *et al*., 2009; Boubakri *et al*., 2010).

In this work, we report that *rep uvrD recF* cells grow poorly on rich medium at low temperature and we isolated five mutations suppressing this LB-cryo-sensitivity. Similarly to the mutations that suppress the growth defects of *rep uvrD* mutants at 37°C, the suppressor mutations isolated here in *rep uvrD recF* mutants at 30°C map in *rpoB* and *rpoC*. One of these mutations is close to the active site; the others are in, or close to, the primary DNA–RNA binding channel, suggesting that they affect the stability of transcription complexes on DNA. Only one of these mutations exhibits a stringent-like phenotype, showing that our assay provides a new way of isolating RNA Pol mutants that are weakly bound to DNA, in transcription initiation or elongation complexes. Furthermore, these RNA Pol mutants allow us to extend our study of helicases that assist replication progression across transcription obstacles.

## Results

### *rep uvrD recF* mutants are cryo-sensitive

We constructed *rep uvrD recF* triple mutants in the presence of a conditional plasmid that carries the *rep* wild-type gene (pAM-rep; Lestini and Michel, 2008). This plasmid only replicates in the presence of the Lac promoter inducer, IPTG and is cured upon cell propagation in the absence of IPTG. We analysed the properties of *rep uvrD recF* cells cured of pAM-rep. For historical reasons the work was realized part in an AB1157 background (classically used for homologous recombination studies) and part in an MG1655 background (the more generally used, sequenced wild-type strain). In the AB1157 background, colony formation was delayed on LB at 37°C and 30°C; at 25°C only a variable subpopulation of cells formed colonies (JJC4048, Table 1). In the MG1655 background, results were similar except for a partial defect of plating efficiency on LB at 30°C (JJC5136 and JJC5166 Table 1). Finally, we constructed an Hfr strain, which allowed the co-introduction of the three *rep uvrD recF* mutations by conjugation, and observed that in this Hfr-PK3-PO131 background the growth defect was more pronounced than in other backgrounds, as plasmid-less *rep uvrD recF* cells were not recovered with the expected efficiency even on minimal medium (MM) at 37°C (not shown). In order to understand the reasons for the cryo-sensitivity of *rep uvrD recF* mutants, we studied five AB1157 *rep uvrD recF* suppressed clones that are able to form large colonies on LB at 30°C in 2 days. The *rpoC*^Δ215–220^ mutation, previously shown to restore the viability of *rep uvrD* cells at 37°C (Boubakri *et al*., 2010), and a suppressor mutation identified in the Hfr *rep uvrD recF* context at 37°C were included in this study.

**Table 1:** *rpoB^sup^* and *rpoC^sup^* mutations suppress the LB-cryo-sensitivity of *rep uvrD recF*, *rep uvrD recO* and *rep uvrD recQ*.

		37°C		30°C	25°C
Strain	Relevant genotype	MM casa	LB	LB	LB
JJC4048	*rep uvrD recF*	1.310^9^ ± 3.710^8^	*7.310^8^* ± *510^8^*	*810^8^* ± *2.210^8^*	< 10^7^
JJC4038/JJC4162	*rep uvrD recF rpoC*^P451L^	1.110^9^ ± 2.510^8^	2.210^9^ ± 210^9^	9.810^8^ ± 8.110^8^	6.410^8^ ± 1.110^8^
JJC4039/JJC4170 JJC4053/JJC4340	*rep uvrD recF rpoC*^H113R^	1.010^9^ ± 1.710^8^	1.110^9^ ± 3.910^8^	9.310^8^ ± 410^8^	7.710^8^ ± 3.610^8^
JJC4040/JJC4163	*rep uvrD recF rpoB*^H447R^	8.110^8^ ± 1.210^8^	8.710^8^ ± 4.510^8^	1.110^9^ ± 5.610^8^	1.110^9^ ± 6.210^8^
JJC4041/JJC4164	*rep uvrD recF rpoB*^D444G^	8.310^8^ ± 2.110^8^	8.510^8^ ± 3.110^8^	8.210^8^ ± 6.710^8^	8.810^8^ ± 7.610^8^
JJC4186/JJC4200	*rep uvrD recF rpoB*^N518D^	6.210^8^ ± 110^8^	8.710^8^ ± 2.310^8^	7.310^8^ ± 2.510^8^	6.310^8^ ± 2.110^8^
JJC1706-S/JJC2488-S	*rep uvrD recO*	1.110^9^ ± 7.910^8^	*6.710^8^* ± *2.610^8^*	*5.410^8^* ± *1.110^8^*	< 10^7^
JJC4283	*rep uvrD recO rpoC*^P451L^	9.810^8^ ± 2.210^8^	8.610^8^ ± 1.910^8^	5.310^8^ ± 5.210^8^	6.110^8^ ± 1.710^8^
JJC4344	*rep uvrD recO rpoC*^H113R^	1.310^9^ ± 510^8^	1.210^9^ ± 2.910^8^	1.410^9^ ± 1.110^8^	1.610^9^ ± 6.610^8^
JJC4342	*rep uvrD recO rpoB*^H447R^	7.710^8^ ± 2.110^8^	7.310^8^ ± 2.510^8^	510^8^ ± 210^8^	5.110^8^ ± 1.910^8^
JJC4324	*rep uvrD recO rpoB*^D444G^	1.210^9^ ± 8.310^8^	1.310^9^± 9.810^8^	1.310^9^ ± 110^9^	1.310^9^ ± 1.110^9^
JJC4257	*rep uvrD recO rpoB*^N518D^	9.110^8^ ± 3.410^8^	6.810^8^ ± 1.310^8^	6.610^8^ ± 1.810^8^	5.610^8^ ± 2.510^8^
JJC5261	*rep uvrD recO rpoC*^Δ215–220^	310^8^ ± 110^8^	7.510^8^ ± 8.710^7^	6.110^8^ ± 1.110^7^	6.810^8^ ± 2.910^7^
JJC3122-S	*rep uvrD recQ*	5.810^8^ ± 1.310^8^	*3.210^7^* ± *2.310^7^*	3.110^6^ ± 3.310^6^	< 10^7^
JJC4902/JJC5313	*rep uvrD recQ rpoC*^P451L^	1.110^9^ ± 2.510^8^	8.210^8^ ± 3.910^8^	6.610^9^ ± 4.610^8^	9.610^8^ ± 4.310^8^
JJC4906	*rep uvrD recQ rpoC*^H113R^	110^9^ ± 4.210^8^	1.110^9^ ± 2.110^8^	1.210^9^ ± 1.310^8^	1.110^9^ ± 2.210^8^
JJC4904	*rep uvrD recQ rpoB*^H447R^	8.910^8^ ± 3.410^8^	8.610^8^ ± 2.510^8^	7.610^8^ ± 1.710^8^	8.310^8^ ± 1.910^8^
JJC4903	*rep uvrD recQ rpoB*^D444G^	610^8^ ± 1.410^8^	7.510^8^ ± 1.910^8^	7.210^8^ ± 1.410^8^	6.810^8^ ± 2.210^8^
JJC4901/JJC5309	*rep uvrD recQ rpoB*^N518D^	7.210^8^ ± 1.510^8^	7.110^8^ ± 1.810^8^	*5.110^8^* ± *1.810^8^*	4.210^6^ ± 3.910^6^
JJC4905	*rep uvrD recQ rpoC*^Δ215–220^	7.310^8^ ± 5.310^7^	7.810^8^ ± 2.610^7^	8.510^8^ ± 6.610^7^	9.810^8^ ± 2.310^8^
JJC5136^a^/JJC5166^a^	*rep uvrD recF*	6.510^8^ ± 1.110^8^	*2.810^8^* ± *1.710^8^*	*1.710^7^* ± *5.810^6^*	< 10^7^
JJC5152^a^/JJC5153^a^	*rep uvrD recF rpoC*^Δ215–220^	6.510^8^ ± 1.910^8^	6.310^8^ ± 1.510^8^	6.610^8^ ± 1.810^8^	5.110^8^ ± 8.410^7^
JJC2451 (pEM001)-S	*rep uvrD recF*[pEM001]	4.210^8^ ± 910^7^	*2.210^8^* ± *110^8^*	< 10^7^	< 10^7^

a Context MG1655. All other strains are in an AB1157 context.

Colonies were counted after 24 h incubation (LB 37°C), 48 h incubation (MM 37°C and LB 30°C), or 3 days incubation (MM 30°C and LB 25°C). Numbers in italics indicate the formation of small colonies appearing 24 h later than wt.

JJCn-S indicates that the strain JJCn was used after segregation of the Rep encoding plasmid. A fresh plasmid-less colony was used for each experiment and cured clones were not kept.

pEM001 is a plasmid that overexpresses RNase H.

### Suppressors of *rep uvrD recF* LB-cryo-sensitivity map in *rpoC* and *rpoB* genes

Analysis of the subpopulation of Hfr plasmid-less colonies formed in 2 days at 37°C on MM supplemented with casamino acids revealed that one of them was resistant to rifampicin. Rif^R^ mutations map in *rpoB*, the gene coding for the β subunit of RNA Pol, which suggested that this particular *rep uvrD recF* clone carries an *rpoB* mutation that suppresses the *rep uvrD recF* growth defect (JJC4100, Table S1). To ascertain that the Rif^R^ mutation was responsible for the improved growth of the *rep uvrD recF* Rif^R^ clone, pAM-rep was reintroduced and the suppressed clone was P1 transduced with *thiC*::Tn*10*, a locus close to the *rpoB rpoC* genes. As expected, the Rif resistance phenotype was 90% linked with *thiC*::Tn*10* (43/48 Tet^R^ transductants were Rif^S^). Curing of pAM-rep showed that *rep uvrD recF thiC*::Tn*10* clones that had remained Rif^R^ had kept the capacity to form colonies overnight at 37°C on LB whereas the *thiC*::Tn*10* clones that had lost the Rif^R^ phenotype grew as poorly as the original Hfr *rep uvrD recF* mutant. This mutation is therefore necessary and sufficient for the improved viability of *rep uvrD recF* cells. Sequencing of the *rpoB* gene revealed the presence of a mutation, N518D, in the *rpoB* Rif^R^ cluster 1.

Overnight cultures of AB1157 *rep uvrD recF* cells were plated on LB at 30°C. Five colonies isolated in three independent experiments were kept for further studies (called S1, S2, and S3a, S3b, S3m). Replacement in the five suppressed strains of the *rep uvrD recF* region of AB1157 by that of the Hfr JJC4100, or of the *rep*::Ap^R^ allele by a *rep*::Cm^R^ allele, did not modify their growth properties (Table S1, data not shown). To determine whether the isolated mutations map in the *rpoBC* genes, a Tet^S^ derivative of each suppressed clones was P1 transduced with *thiC*::Tn*10*. For each mutant 7–11 out of 12 *thiC*::Tn*10* transductants became cryo-sensitive, indicating that these five suppressor mutations are linked to *thiC*.

A plasmid carrying an IPTG-inducible *rpoB*^+^ gene was introduced in Ap^S^ derivatives of the suppressed clones and viability was measured in the presence of IPTG (Table S2). Expression of RpoB rendered S3m cryo-sensitive on LB, suggesting that this mutant carries a recessive *rpoB* mutation; sequencing *rpoB* showed the presence of a H447R mutation. In S3a, the control vector pUC19 could not be introduced whereas the plasmid pUC-*rpoB*^+^ transformed with a normal efficiency (not shown); nevertheless, transformants remained cryo-resistant (Table S2). Sequencing *rpoB* revealed the presence of a D444G mutation. These observations suggest that, in addition to suppressing the *rep uvrD recF* growth defect, the *rpoB*^D444G^ mutation somehow prevents pUC propagation. This defect in plasmid propagation is recessive (as it is complemented in *cis* by the wild-type *rpoB*^+^ allele) whereas the suppression of *rep uvrD recF* cryo-sensitivity is dominant over the wild-type allele. In S1 and S3b expression of *rpoB*^+^ did not prevent growth at 25°C (Table S2), so we sequenced *rpoC* in these two mutants and found a P451L mutation (S1), and a H113R mutation (S3b). Finally, both *rpoB* and *rpoC* genes were sequenced in S2. *rpoB* was intact and *rpoC* carried the H113R mutation, which was thus obtained twice independently. To our knowledge, the three *rpoB*^D444G^, *rpoC*^H113R^ and *rpoC*^P451L^ mutations have not been described previously, whereas the *rpoB*^N518D^ allele has been already isolated in a screen for Rif^R^ clones (Garibyan *et al*., 2003), and the *rpoB*^H447R^ allele in a screen for mutations that increase the expression of amino acid biosynthetic genes in a *relA spoT* context (Trautinger and Lloyd, 2002).

### The *rpo^sup^* mutations are necessary and sufficient for the suppressor phenotype

To determine whether the *rpo* mutations isolated here (called *rpo^sup^* thereafter) are necessary and sufficient for the suppressor phenotype, these mutations were transferred to a *rep uvrD recO* strain by P1 co-transduction with *thiC*::Tn*10*[RecF is known to act in conjunction with two other proteins, RecO and RecR (Kuzminov, 1999), and inactivation of either the RecF, RecO or RecR protein allows colony formation of *rep uvrD* cells at 37°C on MM with casamino acids (Petit and Ehrlich, 2002)]. *rep uvrD recO* cells harbouring the plasmid pBGts-*rep* were used for strain construction; this plasmid carries the wild-type *rep* gene and can be cured by growing cells at 42°C (Petit and Ehrlich, 2002). The phenotype of plasmid-less cells obtained after propagation at 42°C was analysed. The five *rep uvrD recO rpo^sup^* mutants formed about 100% colonies on LB at 37°C, 30°C and 25°C, as the original *rep uvrD recF rpo^sup^* cells (Table 1). We conclude that the *rpo^sup^* mutations are necessary and sufficient to restore full viability to *rep uvrD recF* (*recO*) cells at low temperatures.

RecQ acts in concert with RecFOR to promote RecA binding to blocked forks in the *rep uvrD* mutant (Lestini and Michel, 2008). We observed that the *rep uvrD recQ* mutant is more sensitive to LB than *rep uvrD recF* (*recO*) cells (Table 1), suggesting that the RecFOR proteins still bind to arrested forks in a *rep uvrD recQ* mutant. The *rpo^sup^* alleles suppressed the LB-sensitivity of *rep uvrD recQ* cells, with the notable exception of the Rif^R^ (*rpoB*^N518D^) mutation that suppressed only at 37°C (Table 1). The residual cryo-LB-sensitivity of the *rep uvrD recQ rpoB*^N518D^ indicates that RecFOR bind blocked replication forks in the absence of RecQ in this particular RNA Pol mutant, and that this mutation is a less efficient suppressor than the others at low temperature, in agreement with its original isolation at 37°C. Nevertheless, all other *rpo^sup^* mutations and the *rpoC*^Δ215–220^ mutation suppress the residual growth defects of *rep uvrD recQ* cells.

### Suppression of the LB-cryo-sensitivity of *rep uvrD recF* does not correlate with a stringent-like phenotype of these RNA Pol mutants

**Fig. 1:**
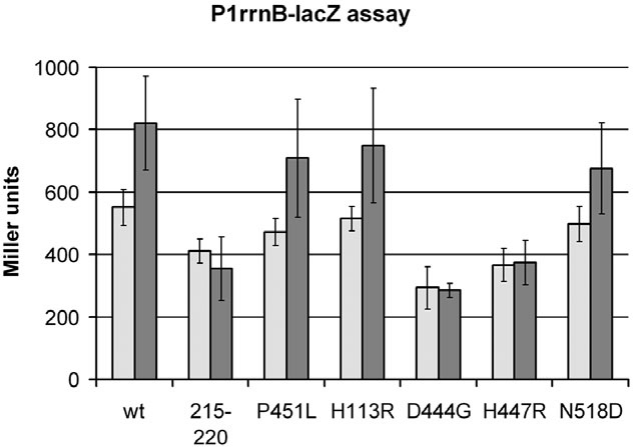
Two of the five *rpo^sup^* RNA Pol mutations affect *rrn* expression in LB. β-galactosidase assays were performed on strains carrying a P_1_*_rrn_*_B_-lacZ fusion and an *rpo^sup^* mutation. The height of the histograms indicates β-galactosidase Miller Units, vertical bars indicate standard deviations. Wt stands for wild-type, Δ215–220 is the control *rpoC*^Δ215–220^ mutation. P451L and H113R are *rpoC* mutations, D444G, H447R and N518D are *rpoB* mutations. Light grey: cells grown in MM; dark grey: cells grown in LB.

The *rpoC*^Δ215–220^ mutation alters the kinetic properties of transcription complexes, reducing rRNA transcription and increasing transcription from some amino acid biosynthetic genes (Bartlett *et al*., 1998). On the other hand, it behaves as most of the mutations isolated here as it suppresses the growth defects of *rep uvrD recF*, *recO* and *recQ* cells at all temperatures (Boubakri *et al*., 2010) (Table 1). In addition, one of the mutations isolated here (*rpoB*^H447R^) was also previously isolated in a screen for mutations that confer a stringent-like phenotype (Trautinger and Lloyd, 2002). These observations prompted us to test whether the *rpo^sup^* mutations isolated here prevent the stimulation of the *rrnB* core promoter after a shift to rich medium. The *rpo^sup^* mutations were co-transduced with *thiC*::Tn*10* into a strain carrying a P1*_rrnB_*-*lacZ* fusion, which was used to compare the expression of the P1*_rrnB_* core promoter in MM and in LB (Bartlett *et al*., 1998). As expected, in wild-type cells the expression of *lacZ* from P1*_rrnB_* promoter was higher in LB than in MM, whereas P1*_rrnB_* activity remained low in both media in the presence of the *rpoC*^Δ215–220^ or *rpoB*^H447R^ mutations (Fig. 1). This experiment revealed that *rpoB*^D444G^ also reduces rRNA expression in LB (Fig. 1). In contrast, P1*_rrnB_* expression remained higher in LB than in minimal medium in the presence of the *rpoB*^N518D^, *rpoC*^P451L^ and *rpoC*^H113R^ alleles as in wild-type cells, showing that none of these three mutations affects the activity of rRNA promoter.

**Fig. 3:**
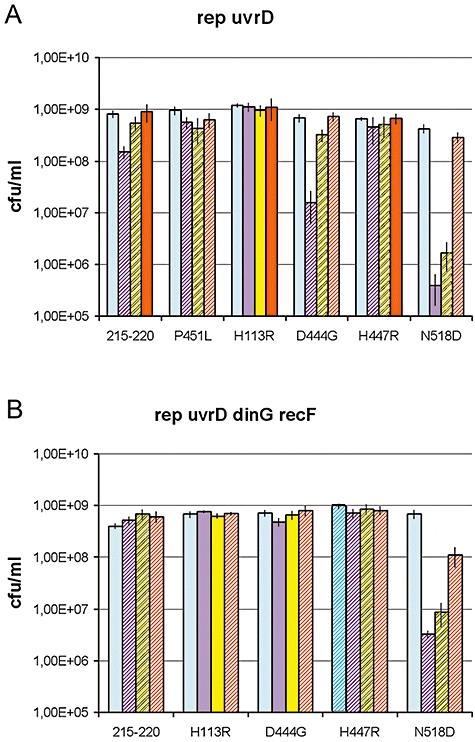
A. *rpo^sup^* RNA Pol mutations restore growth of *rep uvrD* cells. The height of the histograms indicates the number of colony-forming units (cfu) per ml, vertical bars indicate standard deviations. Rpo wild-type *rep uvrD* cells are not shown because they are lethal in all these conditions (plating on MM with casamino acids or on LB, at 37°C or 30°C). Mutants are in the AB1157 context, similar results were previously published for the *rep uvrD rpoC*^Δ215–220^ mutant at 37°C in the MG1655 context (*Boubakri et al.*, 2010). Δ215–220 and H113R are *rpoC* mutations, D444G, H447R and N518D are *rpoB* mutations. Light blue: MM 30°C (plating efficiencies on MM 37°C are not shown and were similar to those at 30°C); purple: LB 25°C; yellow: LB 30°C; orange: LB 37°C. Full boxes: colonies formed in 24 h (37°C LB), 48 h (30°C LB), or 3 days (30°C MM, 25°C LB). Hatched boxes: colonies appearing 24 h later than these normal times. B. Three *rpo^sup^* RNA Pol mutations restore growth of *rep uvrD dinG recF* cells at all temperatures. Rpo wild-type and *rpoC*^P451L^ cells are not shown because they are lethal under these conditions (plating on MM or on LB, at 37°C or 30°C). Mutants are in the MG1655 context, similar results were obtained in the AB1157 context (not shown). Results for the *rep uvrD dinG recF rpoC*^Δ215–220^ mutant at 37°C were previously published (Boubakri *et al*., 2010), and were reproduced here as a control. Plating efficiencies on MM 37°C are not shown and were similar to those at 30°C.

RelA and SpoT proteins are implicated in ppGpp alarmone synthesis. *relA spoT* double mutants do not induce the stringent response and are thus unable to grow on MM unless amino acids are provided. RNA Pol mutations that mimic the stringent response are classically isolated as suppressing the auxotrophy of *relA spoT* mutants. Although this has not been actually demonstrated, it was suggested that the inability of *relA spoT* mutants to grow on MM and the ability of the *rpoBC* suppressor mutations to suppress this defect results from effects of the mutations on transcription of amino acid biosynthetic operons (Paul *et al*., 2005; see Rutherford *et al*., 2009, for discussion). In order to analyse the capacity of the *rpo^sup^* mutations to suppress the auxotrophy of *relA spoT* double mutants, we constructed *rpo^sup^*Δ*relA*::Kan^R^ mutants (strains JJC4553 to JJC4559 Table S1) and P1-transduced them with a Δ*spoT*::Cm^R^ null mutation, plating half of the transduction mixture on LB and half on MM. All *rpo^sup^*Δ*relA*::Kan^R^ mutants could be transduced by the Δ*spoT*::Cm^R^ null mutation on LB while only one mutant, *rpoB*^H447R^Δ*relA*::Kan^R^, provided transductants on MM (Table S3). As expected from this result, none of the *rpo^sup^*Δ*relA*::Kan^R^Δ*spoT*::Cm^R^ mutants obtained on LB could grow on MM except for the *rpoB*^H447R^Δ*relA*::Kan^R^Δ*spoT*::Cm^R^ strain (Table S3). The growth defect on MM was specific for a *relA spoT* context, because none of the RNA Pol mutations prevented growth on MM in RelA^+^ SpoT^+^ cells (see plating efficiencies on MM in Figs 3 and 4). This result shows that only the *rpoB*^H447R^ mutation prevents the increase of *rrn* expression in rich medium and allows growth of a *relA spoT* mutant on MM. The *rpoB*^D444G^ mutation only affects *rrn* expression and the other *rpo^sup^* mutations exhibit none of these stringent-like phenotypes. This result shows that four of the five mutations isolated in this study would not be obtained in a classical screen for mutations that restore growth of a *relA spoT* mutant on MM. Although they do not confer a stringent-like phenotype, the *rpo^sup^* mutations bypass the need for accessory replication helicases, suggesting that they affect the stability of transcription complexes without affecting their kinetic properties on *rrn* and amino acid biosynthetic gene promoters. The possible instability of the mutant RNA Pol–DNA complexes was tested by two different genetic approaches.

### *rpo^sup^* mutations improve the resistance to UV irradiation of a *ruv* mutant and the *rpoC*^H113R^ mutation rescues a *greA greB* double mutant

**Fig. 2:**
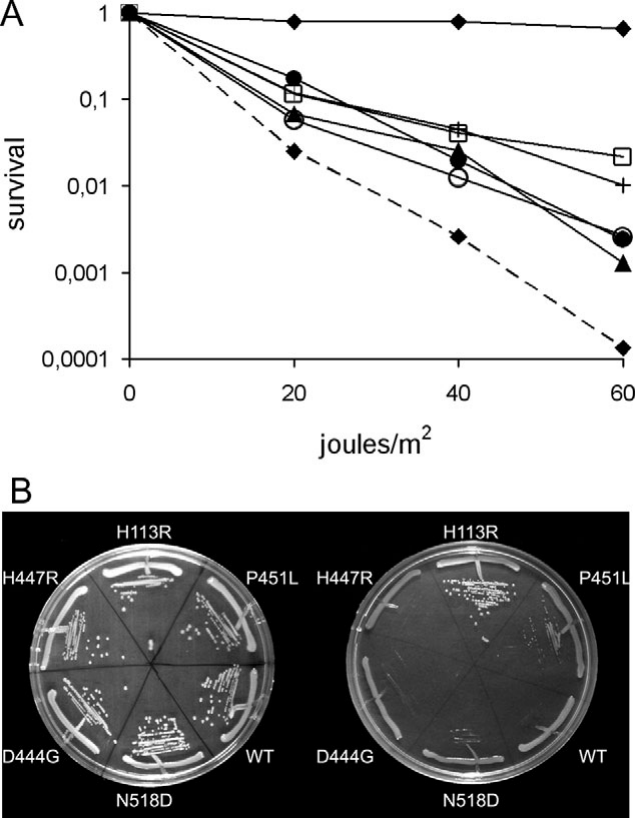
A. Four *rpo^sup^* RNA Pol mutations improve growth of UV-irradiated *ruvABC* mutants. Survival of UV-irradiated cells, results are the average of three to six independent determinations. Wild-type cells (JJC40) diamonds full line, *ruvABC* (JJC754) diamond dashed line, *rpoC*^Δ215–220^*ruvABC* (JJC4886) crosses, *rpoC*^P451L^*ruvABC* (JJC4548) open square, *rpoB*^H447R^ *ruvABC* (JJC4832) closed circle, *rpoB*^D444G^ *ruvABC* (JJC4549) open circles, *rpoB*^N518D^ *ruvABC* (JJC4547) triangles. B. The *rpoC*^H113R^ mutation restores the viability of the *ΔgreA*::Cm^R^*ΔgreB*::Kan^R^ double mutant at 42°C. *ΔgreA*::Cm^R^*ΔgreB*::Kan^R^*rpo^sup^* strains were streaked on LB Cm plates at 30°C (left) and at 42°C (right). Top sector, *greA greB rpo*^CH113R^ (JJC4923) the only thermoresistant *greA greB* double mutant. Turning in the clockwise direction from this mutant: *greA greB rpoC*^P451L^ (JJC5456), *greA greB* Rpo wt (JJC5455), *greA greB rpoB*^N518D^ (JJC4818), *greA greB rpoB*^D444G^ (JJC4820), *greA greB rpoB*^H447R^ (JJC4821).

RuvABC is a recombination complex that acts at the last step of homologous recombination by resolving recombination intermediates called Holliday junctions (Kuzminov, 1999). *ruv* mutants are hypersensitive to UV irradiation and *rpoB* or *rpoC* mutations that exhibit a stringent-like phenotype partially relieve this hypersensitivity. It was proposed that they increase the intrinsic instability of RNA Pol–DNA complexes when RNA Pol is blocked by a DNA lesion (Trautinger and Lloyd, 2002). A *ruvABC* deletion was introduced in all *rpo^sup^* mutants and the UV resistance of the resulting strains was measured. With the exception of the *rpoC*^H113R^ allele, which was poorly viable in a *ruv* mutant context and yielded variable results, all mutations improved the UV resistance of the *ruvABC* mutant (Fig. 2A). This result supports the idea that the mutations isolated here affect the stability of RNA Pol–DNA complexes, either at promoters or in TEC.

When RNA Pol encounters a block during elongation and backtracks, the transcription factors GreA and GreB suppress pausing by stimulating the intrinsic nucleolytic activity of RNA Pol (reviewed in Borukhov *et al*., 2005). *greA greB* double mutants are non-viable at high temperature, presumably because prolonged RNA polymerase pausing prevents replication and/or transcription. A mutation isolated through its stringent-like phenotype was previously reported to suppress the thermo-sensitivity of *greA greB* mutants (Trautinger and Lloyd, 2002). *greA greB rpo^sup^* mutants were constructed and tested for growth at 42°C. Only the *rpoC*^H113R^ mutation allowed the growth of *greA greB* mutant at 42°C (Fig. 2B). This result presumably reflects the destabilization of backtracked RNA Pol by the *rpoC*^H113R^, but this level or type of destabilization is not essential for the growth of *rep uvrD recF* cells at 30°C as it is observed for only one of the suppressor mutations. This result also indicates that mutations that do not affect *rrn* or amino acid biosynthetic gene expression can nevertheless decrease the stability of stalled TEC enough to rescue a *greA greB* mutant. Altogether, we infer from the phenotypes conferred by the RNA Pol mutations described here that these mutations compromise the stability of transcription complexes; the consequences of this putative destabilization were further investigated in backgrounds that lack different accessory helicases.

### Several *rpo^sup^* mutants rescue *rep uvrD* mutants in a RecF^+^ context

If the viability of *rep uvrD recF rpo^sup^* mutants results from less replication arrest, or from facilitated replication restart, the inactivation of *recF* might not be needed for viability. Actually, certain RNA Pol mutations including the *rpoC*^Δ215–220^ allele were reported to suppress the growth defect of *rep uvrD* cells in a RecF^+^ context at 37°C (Guy *et al*., 2009; Boubakri *et al*., 2010). To construct *rep uvrD rpo^sup^* mutants, *rep* and *uvrD* were introduced in *rpo^sup^* single mutants containing pGBts-*rep*, and/or the *rpo^sup^* mutation was co-transduced with *thiC*::Tn*10* to a *rep uvrD* (pAM-rep) mutant. The viability of plasmid-less clones was measured after curing cells by propagation at 42°C (pGBts-rep) or in the absence of IPTG (pAM-rep) (Fig. 3A). The *rpoC*^H113R^ mutation allowed formation or *rep uvrD* colonies on LB at all temperatures. The *rpoC*^P451L^ mutant yielded slow-growing colonies that were heterogeneous in size at low temperature. Two of the mutations that affect *rrn* expression (*rpoC*^Δ215–220^, *rpoB*^H447R^) restored *rep uvrD* cells viability on LB, but colony formation was delayed at low temperature and also slightly decreased for *rpoC*^Δ215–220^ at 25°C. In the *rpoB*^D444G^ context, colony formation on LB was delayed at all temperatures and significantly decreased at 25°C. Finally, the *rpoB*^N518D^ mutation had only a partial effect at 37°C and did not allow colony formation at low temperatures. These observations indicate that in one *rep uvrD rpo^sup^* mutant (*rpoC*^H113R^) RecFOR does not bind replication forks at any temperature. In the other *rep uvrD rpo^sup^* mutants, although RecFOR is not lethal any more as in *rep uvrD* Rpo^+^ cells, it remains partly deleterious, slowing down and/or preventing growth, mainly at low temperature. Therefore, the modifications of RNA Pol activity caused by the different mutations determine the accessibility of replication forks to RecFOR recombination proteins.

### *Rpo^sup^* mutations rescue *rep uvrD recF dinG* mutants but not *rep uvrD dinG* mutants

The viability of the *rep uvrD recF* mutant relies on the presence of a third helicase called DinG (Boubakri *et al*., 2010). We previously reported that the *rpoC*^Δ215–220^ mutation suppresses the lethality of *rep uvrD recF dinG* mutants at 37°C on MM and on LB, but not that of *rep uvrD dinG* mutants, indicating that it facilitates replication restart in the absence of all three accessory helicases only if RecF does not poison arrested forks (Boubakri *et al*., 2010). We tested whether the *rpo^sup^* mutations also suppress *rep uvrD recF dinG* lethality by constructing *rep uvrD recF dinG rpo^sup^* mutants in two ways, first a *dinG* deletion was introduced in the original *rep uvrD recF rpo^sup^* clones (JJC4043 to JJC4047, context AB1157, Table S1), and second the *rpo^sup^* mutations were introduced in a *rep uvrD recF dinG* mutant (JJC5405 to JJC5426, context MG1655, Table S1). Results were similar in both backgrounds (data not shown and Fig. 3B). *rpoC*^P451L^ was the only mutation that did not suppress the lethality of *rep uvrD recF dinG* cells as no plasmid-less cells could be obtained (not shown). The best suppressor mutation was again the *rpoC*^H113R^, which restored 100% *rep uvrD dinG recF* plating efficiency at 37°C (although colony formation was delayed) and at low temperature (Fig. 3B). The three mutations *rpoC*^Δ215–220^, *rpoB*^H447R^ and *rpoB*^D444G^ also restored 100% plating efficiency but colony formation was delayed on LB at 37°C for *rpoB*^D444G^, on LB at all temperatures for *rpoC*^Δ215–220^, and in all growth conditions for *rpoB*^H447R^. Finally, *rpoB*^N518D^ allowed normal colony formation on MM but formed only about 10% of heterogeneous slow-growing colonies on LB at 37°C and did not suppress the *rep uvrD recF dinG* lethality on LB at low temperature.

It was previously reported that *rpoC*^Δ215–220^ only rescues a *rep uvrD dinG* mutant that lacks RecF (Boubakri *et al*., 2010). To address this question for the *rpo^sup^* mutants, *rep uvrD dinG rpo^sup^* RecF^+^ mutants were constructed, first by introducing the *dinG* deletion in the original *rep uvrD rpo^sup^* mutants previously made RecF^+^ (JJC5253 to JJC5257 context AB1157 and JJC5258 context JJC4100, Table S1) and second by introducing successively all three helicase deletions in the *rpo^sup^* mutants (JJC4911 to JJC4918, JJC5310 and JJC5311, context AB1157, Table S1). All strains yielded similar results: the *rpo^sup^* mutations did not suppress the lethality of *rep uvrD dinG* mutants in a RecF^+^ context, as either no plasmid-less colonies could be recovered after growth under non-permissive conditions for plasmid replication (*rpoB*^N518D^), or only a few plasmid-less colonies were obtained that could not be propagated and eventually acquired additional suppressive mutations, possibly because the *uvrD* context is mutator (data not shown). In conclusion, in the absence of all three helicases the *rpo^sup^* mutations restore cell viability only if *recF* is inactivated, as previously observed for the *rpoC*^Δ215–220^ mutation in a MG1655 background at 37°C (Boubakri *et al*., 2010). This result indicates that, at least in the absence of the three Rep, UvrD and DinG helicases, replication forks are still arrested by the encounter of the *rpo^sup^* mutated RNA polymerases, allowing RecFOR to gain access to DNA. This result also indicates that DinG is responsible of the viability of *rep uvrD rpo^sup^* DinG^+^ mutants shown in Fig. 3A, as the inactivation of *dinG* in this context is lethal.

To further test the effects of the *rpo^sup^* mutations on replication–transcription collisions, we introduced these mutations in cells where such collisions are increased by a chromosome rearrangement.

### Rescue of helicase mutants that carry an inverted *rrn* operon depends on the *rpo* mutation

**Fig. 4:**
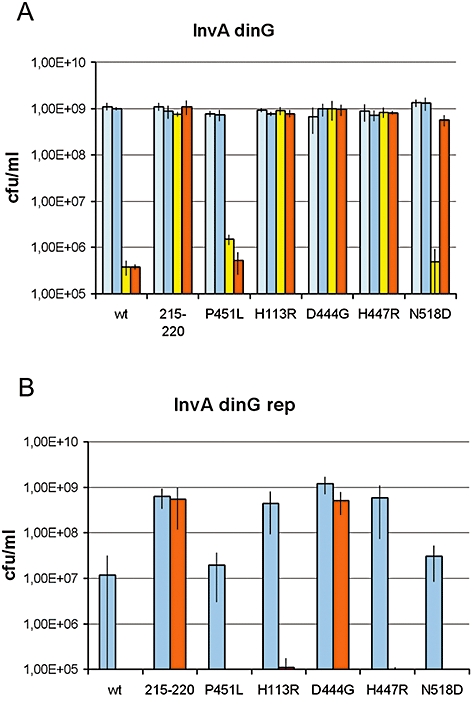
A. Three *rpo*^sup^ RNA Pol mutations restore growth of InvA *dinG* cells at all temperatures. The height of the histograms indicates the number of cfu per ml, vertical bars indicate standard deviations. Results for the InvA *dinG rpoC*^Δ215–220^ mutant at 37°C were previously published (Boubakri *et al*., 2010), and were reproduced here as a control. Δ215–220, P451L and H113R are *rpoC* mutations, D444G, H447R and N518D are *rpoB* mutations (MG1655 context). Symbols are as in Fig. 3, dark blue are plating efficiencies on MM at 37°C. B. Three *rpo^sup^* mutations restores growth of InvA *dinG rep* cells at 37°C on MM, only *rpoB*^D444G^ also restores viability on LB. Results for the InvA *rep dinG rpoC*^Δ215–220^ mutant were previously published (Boubakri *et al*., 2010), and were reproduced here as a control.

Inversion of an *rrn* operon creates a region of increased head-on collisions between replication and transcription. Such inversions render the *dinG* mutant sensitive to rich medium because of R-loop formation, and strongly impair growth of the *rep dinG* double mutant, even on MM, because of DNA Pol–RNA Pol collisions (Boubakri *et al*., 2010) (Fig. 4A). In a strain that carries an inverted *rrnA* operon (InvA) the *rpoC*^Δ215–220^ mutation suppresses the growth defects of both *dinG* and *dinG rep* mutants at 37°C, on MM and on LB, presumably by decreasing the transcription efficiency of *rrnA* (Boubakri *et al*., 2010) (Fig. 4A).

We tested whether the *rpo^sup^* mutations decrease the formation of R-loops in inverted *rrn* by introducing these mutations in InvA *dinG* cells and measuring plating efficiencies of the resulting combination of mutations on LB*. rpoC*^Δ215–220^, previously tested at 37°C only, was tested here at 30°C. The *rpoC*^H113R^ mutation and the three mutations that affect *rrn* expression (*rpoB*^H447R^*rpoB*^D444G^ and *rpoC*^Δ215–220^) restored full viability of the InvA *dinG* mutant at 37°C and 30°C (Fig. 4A). Therefore, they decrease R-loop formation in this context at both temperatures. In contrast the Rif^R^ mutation (*rpoB*^N518D^) allowed InvA *dinG* colony formation on LB at 37°C but not at 30°C, and the *rpoC*^P451L^ mutations had no effect. The phenotype of this last mutant suggests that suppression of the *rep uvrD recF* LB-cryo-sensitivity by *rpo^sup^* mutations is independent of R-loop removal. To ascertain directly that the LB-cryo-sensitivity of the *rep uvrD recF* mutant does not result from R-loop formation, a plasmid that overexpresses RnaseH (pEM001) was introduced in this mutant. As shown in Table 1, this plasmid did not improve plating efficiency. In conclusion, most of the *rpo^sup^* mutations suppress or decrease R-loop formation in InvA cells, allowing InvA *dinG* viability, but conversely suppression of R-loops is neither necessary nor sufficient to improve growth of *rep uvrD recF* cells at 30°C on LB.

*rpo^sup^* mutations were also introduced in an InvA *dinG rep* mutant, where replication is arrested in the inverted *rrnA* by collisions with RNA Pol (Boubakri *et al*., 2010). As previously reported the mutation *rpoC*^Δ215–220^ restored a normal plating efficiency on MM and LB (Boubakri *et al*., 2010), and as expected the *rpoB*^D444G^ mutation, which decreases P*rrn* efficiency (Fig. 1), exhibited a similar phenotype (Fig. 4B). Surprisingly, the *rpoB*^H447R^ mutation improved colony formation only on MM. It is conceivable that the decrease of *rrn* expression measured in Fig. 1 with a promoter deprived of its FIS-binding sites is compensated by the presence of these sites at inverted *rrnA* (Bartlett *et al*., 2000). Finally, *rpoC*^H113R^ also improved viability on MM whereas the *rpoC*^P451L^ and *rpoB*^N518D^ mutations had no effect. Altogether, these results indicate that in rich medium only two mutations, *rpoC*^Δ215–220^ and *rpoB*^D444G^, destabilize RNA Pol enough to allow replication across an inverted *rrn* in the absence of Rep and DinG, i.e. when only the UvrD helicase is active. Among the suppressor mutations that do not affect *rrn* expression, *rpoC*^H113R^ is the only one that rescues an InvA *dinG rep* mutant, and only on MM.

A consequence of replication arrest in the *rep* mutant is a reaction called replication fork reversal, in which the two ends of the newly synthesized strands at a blocked replication fork anneal to form a DNA double-strand end and a Holliday junction. This reaction renders RecBC (the recombination enzyme specific for DNA double-strand ends) essential for viability (Seigneur *et al*., 1998; Michel *et al*., 2007). Interestingly, although this study and other recent studies suggest that in the *rep* mutant replication forks are arrested by transcribed sequences, none of the mutations isolated here rescued the viability of *rep recB* cells, as no plasmid-less colonies could be recovered after propagation of *rep recB rpo^sup^* (pAM-rep) mutants in the absence of IPTG (data not shown, strains JJC5532 to JJC5536 in Table S1). This indicates that the *rpoB* mutation does not prevent replication fork reversal in a *rep* mutant, and that, even when the RNA Pol is mutated and weakly bound to DNA, its removal from DNA by UvrD and/or DinG takes place within the context of restarting reverted replication forks.

## Discussion

We show here that transcription is a stronger obstacle to replication at lower temperatures than at 37°C and we isolated mutations that suppress the rich medium cryo-sensitivity of *rep uvrD recF* cells. The genetic properties of these mutants suggest that the *rep uvrD* and *rep uvrD recF* strains provide a new way of isolating mutations that decrease the stability of RNA Pol on DNA, at promoters and/or in TEC. Furthermore, the analysis of these RNA Pol mutants reinforces the notion that Rep, UvrD and DinG have redundant functions in *E. coli* to facilitate the progression of replication forks across transcribed sequences.

### Isolation and properties of new RNA Pol alleles

**Fig. 5:**
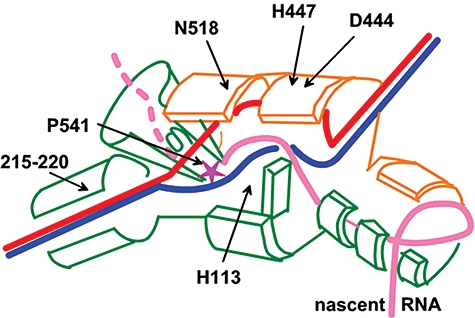
Schematic representation of the RpoB and RpoC subunits of RNA Pol showing the position of the *rpo^sup^* mutations. In orange RpoB, in green RpoC. Blue and red lines represent the template and non-template DNA-strands, respectively, the pink line represents the neo-synthesized RNA (the putative backtracked RNA is shown in dashed pink line). Positions of the *rpo^sup^* mutations are indicated (adapted with permission from Nudler, 2009).

The atomic structure of *Thermus thermophilus* and *Termus aquaticus* RNA Pol have led to structural models of TEC (Zhang *et al*., 1999; Korzheva *et al*., 2000; Vassylyev *et al*., 2002, 2007a,b). The similarity of these RNA Pols with the *E. coli* enzyme allows us to map the residues affected in our *rpoB* and *rpoC* mutants on these structures. The position of the mutated amino acids on a representation of a wild-type transcription complex is shown in Fig. 5. The three *rpoB* mutations, D444G, H447R and N518D (D324, H327 and T398 in *T. thermophilus* and *T. aquaticus*) lie in the major channel for the DNA/RNA hybrid, in, or close to the β-lobe 1, in a region previously described to be important for the stability of RNA Pol–DNA complexes (Trautinger and Lloyd, 2002; Rutherford *et al*., 2009). The *rpoC*^H113R^ mutation (H90 in *T. thermophilus* and H101 in *T. aquaticus*) is on the other side of the major channel and although the mutated residue is buried in the enzyme, it may affect interactions of RpoC with the hybrid DNA-RNA. *rpoC*^P451L^ (P719 in *T. thermophilus* and P730 in *T. aquaticus*) is in a different location in the complex, in a highly conserved region around the active site. This mutation is adjacent to a mutation isolated as a Δ*dksA* suppressor (H450R) and, as the RNA Pol cofactor DksA is also involved in the stability of transcription complexes, mutations in this region could affect the enzyme in another way to those which flank the major channel (Rutherford *et al*., 2009). In spite of the proximity of this mutation to the active site, we did not detect any deleterious effect of the *rpoC*^P451L^ allele on *E. coli* growth (data not shown). Biochemical analysis of these mutant enzymes would tell whether the *in vivo* instability of transcription complexes is related to an increased propensity to pause and/or to terminate transcription.

The phenotypes of the isolated RNA Pol mutations do not allow their classification in specific groups but allow us to rank them according to the advantage that they confer to cells that lack Rep, UvrD and/or DinG helicases (summarized in Table 2). The best suppressor of growth defects in the absence of these helicases is the *rpoC*^H113R^ mutation, which was isolated twice independently. This RNA Pol mutant is the only one that forms TEC unstable enough to suppress the temperature sensitivity of a *greA greB* double mutant. It confers LB resistance to *rep uvrD*, *rep uvrD dinG recF* and InvA *dinG* cells at all temperatures, and improves growth of InvA *dinG rep* cells at 37°C on MM. Of all mutants isolated here, the RpoC^H113R^ RNA Pol is probably the one that forms the weakest complex on DNA. It is also the only one that slows down the growth of otherwise wild-type cells (data not shown), and could not be reliably combined with a *ruvABC* deletion. Slightly less efficient than *rpoC*^H113R^, the mutations *rpoB*^H447R^ and *rpoB*^D444G^ confer an intermediate phenotype, as the previously described *rpoC*^Δ215–220^ allele. The destabilization of transcription complexes caused by these mutations, which is deduced from their stringent-like phenotype and from the increased UV resistance conferred to *ruv* mutants, allows *rep uvrD dinG recF* and *rep uvrD* cells to grow on LB. They rescue InvA *dinG* and InvA *rep dinG* mutants, with the exception of the LB sensitivity of InvA *rep dinG rpoB*^H447R^ cells (discussed below). Finally, the two less efficient suppressors are *rpoC*^P451L^ and *rpoB*^N518D^. *rpoC*^P451L^ is the only mutation that is close to the active site, while *rpoB*^N518D^ was isolated at 37°C. The only sign that these mutated RNA Pols might be weakly bound to DNA, beside the rescue of *rep uvrD recF* cells, is the increase of UV resistance in *ruv* mutants. In their presence, *rep uvrD* cells form slow-growing colonies only at 37°C, and InvA *dinG rep* cells remain sick on MM. Furthermore, *rpoC*^P451L^ fails to rescue InvA *dinG* cells on LB, while *rpoB*^N518D^ only rescues it at 37°C and does not rescue *rep uvrD recQ* cells at 30°C.

**Table 2:** Summary of the phenotypes conferred by the different rpoB/C mutations.

	P1*rrn*B in LB	*relA spoT* on MM	*greA greB* at 42°C	*rep uvrD* RecF^+^ (LB)	*rep uvrD dinG recF* (LB)	InvA dinG (LB)	InvA *dinG rep* (LB)
*rpoBC*+	high	−	−	−	−	−	−
*rpoC*^H113R^	high	−	**+**	**+**	**+**	**+**	−(MM**+**)
*rpoC*^Δ215–220^	low	**+**^a^	−	**+**(∼cryoS)	**+**(delayed)	**+**	**+**
*rpoB*^D444G^	low	−	−	delayed and cryoS	**+**	**+**	**+**
*rpoB*^H447R^	low	**+**	−	**+**(delayed at low T°)	**+**(delayed)	**+**	−(MM**+**)
*rpoC*^P451L^	high	−	−	**+**(delayed)	−	−	−
*rpoB*^N518D^	high	−	−	delayed and cryoS	cryoS	cryoS	−

a Bartlett *et al*., 1998.

In addition to the phenotypes indicated here, all mutations rescue a *rep uvrD recF (recO)* mutant on LB at all temperatures, all rescue a *rep uvrD recQ* mutant on LB (except *rpoB*^N518D^ at low temperature), all improve the UV resistance of a *ruvABC* mutant (except *rpoC*^H113R^ which could not be reliably combined with the *ruvABC* mutation) and none rescue the lethality of a *rep uvrD dinG* triple mutant.

Several mutations that mimic the effects of ppGpp have been isolated by different means, most often because they activate amino acid biosynthetic genes in a *relA spoT* double mutant, enabling it to grow on minimal medium (Bartlett *et al*., 1998; Zhou and Jin, 1998; Trautinger and Lloyd, 2002; Broccoli *et al*., 2004; Szalewska-Palasz *et al*., 2007). Some of these mutations decrease expression from the *rrn* promoter regardless of the presence of the upstream FIS-binding sites (Szalewska-Palasz *et al*., 2007), while the FIS sites compensate for the *rpoC*^Δ215–220^ mutation effects on the *rrnB* promoter (Bartlett *et al*., 2000). One mutation conferring a stringent-like phenotype was shown to reduce R-loop formation in a non-translated sequence by causing premature transcription arrest *in vivo* (Broccoli *et al*., 2004), and two others were shown to decrease the stability of TEC *in vitro* (Trautinger *et al*., 2005). Therefore, the *rpoC*^Δ215–220^, *rpoB*^D444G^ and *rpoB*^H447R^ mutations may be affected for the stability of transcription initiation complexes, or for the stability of TEC, or for both. Because the FIS sites compensate for the *rpoC*^Δ215–220^ mutation effects on the *rrnB* promoter, this mutation is likely to rescue helicase mutants carrying an inverted *rrn* by destabilizing TEC, as proposed in Boubakri *et al*., (2010). *rpoB*^D444G^ may rescue InvA *dinG rep* cells because the promoter remains weakly expressed on LB in the presence of the FIS Sites, or because TEC are unstable. The RpoB^H447R^ RNA Pol may fail to rescue InvA *dinG rep* cells because its defects are compensated for by the presence of the FIS sites and it forms more stable TEC than the RpoC^Δ215–220^ RNA Pol during *rrn* transcription. This observation supports the idea that there is no necessary correlation between the instability of TEC and the instability of the open complexes on *rrn* promoters. Furthermore, we isolated here a mutant (*rpoB*^D444G^) that affects transcription initiation at *rrn* promoters without affecting the expression of amino acids biosynthesis genes in a *relA spoT* context. To our knowledge, such a mutant was not reported before. Finally, rescue of a *greA greB* mutant at high temperature was previously described for a RNA Pol mutation isolated as increasing amino acids biosynthetic genes expression (Trautinger and Lloyd, 2002) whereas here it is observed for a RNA Pol mutant that does not affect these genes, nor *rrn* expression (*rpoC*^H113R^).

We used strains carrying a chromosome inversion to measure the consequences of a weaker stability of transcription complexes when the rate of replication arrest is increased either by the formation of R-loops, or by encountering oppositely oriented highly active RNA Pol in inverted *rrn*. With the exception of *rpoC*^P451L^, all RNA Pol mutations isolated here prevent R-loop formation. This means that RNA Pols that form unstable transcription complexes are less prone than the wild-type enzyme to R-loop formation. Conversely, only one (*rpoB*^D444G^) could prevent the lethality associated with the encounter of a series of RNA Pol transcribing a ribosomal operon in the direction opposite to replication. This means that even RNA Pols weakly bound to DNA will arrest replication forks when the latter collide with an oppositely oriented highly transcribed operon.

### How does *E. coli* deal with replication–transcription collisions?

The general scheme that emerges from the present study and from previous studies of *rep*, *dinG* and *uvrD* single and multiple mutants is that Rep is the first factor that facilitates replication through transcribed sequences *in vivo* (Lane and Denhardt, 1975; Seigneur *et al*., 1998; Petit and Ehrlich, 2002; Guy *et al*., 2009; Boubakri *et al*., 2010)*.* In the absence of Rep, UvrD becomes essential for *E. coli* viability and mutations in RNA Pol suppress this co-lethality, which points to UvrD as the main back-up to Rep for RNA Pol removal (Guy *et al*., 2009; Boubakri *et al*., 2010; this work). Two lines of evidence point to DinG as the other helicase that acts as a back-up. The first is our previous report that even in the absence of RecF, the combination of the *rep, uvrD* and *dinG* mutations is lethal in *E. coli* expressing the wild-type RNA Pol (Boubakri *et al*., 2010). This suggests that DinG removes wild-type RNA Pols from replication forks when Rep, UvrD and RecF are absent. The second, is our present observation that DinG is required for suppression of the *rep uvrD* co-lethality by most of the *rpo^sup^* mutations studied here, indicating that because they are unstable on DNA, the mutant RNA Pol can be removed from replication forks by DinG regardless of the presence of RecF. However, these mutated RNA Pols do not suppress the need for RecBC in a *rep* mutant, indicating that DinG and UvrD act in the context of restarting reversed replication forks. These helicases may recognize some features of reversed or of the restarting replication forks that are not shared by the original replication fork, whereas Rep may act within the context of the original replication fork that encounters the obstacle. It should be noted that the *rpo^sup^* mutations do not prevent spontaneous replication arrest in the *E. coli* chromosome because they did not relieve the growth defects of a *priA* mutant, which lacks the main replication restart protein PriA (strains JJC5540 and JJC5541 in Table S1, data not shown).

At low temperature, even in the presence of DinG, *rep uvrD recF* cells grow poorly on LB, indicating that DinG has a limited ability to remove the wild-type RNA Pol from replication forks under these growth conditions. Either transcription is a stronger obstacle to replication at low temperature, for example because transcriptional complexes are more stable, or for some reason DinG is less active. Provided that RecF is absent, several RNA Pol mutations isolated here bypass not only the need for Rep and UvrD but also the need for DinG (*rep uvrD dinG recF rpo^sup^* are viable, Fig. 3B). Removal of the mutant RNA Pol may then be catalysed either by some yet unknown function or by the replisome itself. Actually, in a purified system *in vitro* the wild-type RNA Pol initiation complex can be dislodged by the replisome (Pomerantz and O'Donnell, 2010). However, as RecF remains lethal in a *rep uvrD dinG rpo^sup^* mutant, replication forks are still arrested in these *rpo^sup^* cells that lack the three helicases, which is another indication that the dislodging of RNA Pol takes place during replication restart.

Our study of RNA Pol mutants that form unstable complexes on DNA provided conditions where viability is dependent on DinG only when RecF is present (the *rep uvrD rpo^sup^* combinations of mutations become LB-sensitive upon DinG inactivation only in a RecF^+^ context). This observation suggests that DinG counteracts a deleterious action of RecF; either DinG could remove RecFOR/RecA from DNA, or it could prevent RecFOR binding. It should be noted that UvrD can act both ways, it removes RecA from DNA or prevents RecFOR binding, depending on the cause of replication arrest (Lestini and Michel, 2007). The hypothetical removal of RecFOR and/or RecA from DNA by DinG needs to be tested *in vitro*; nevertheless, we do not favour this hypothesis because it is difficult to explain why DinG would remove RecF only in certain RNA Pol mutants. We favour the hypothesis that DinG and RecFOR compete for blocked replication forks in *rep uvrD* cells. Because DinG is unable to remove the wild-type RNA Pol, *rep uvrD* cells are killed by RecFOR. Similarly, the *rep uvrD rpo*^N518D^ mutant is non-viable on LB at low temperature because under these conditions DinG cannot dislodge this mutated RNA Pol from DNA, letting RecFOR bind to arrested replication forks. Conversely, DinG is capable of removing all other RNA Pol mutants isolated here before RecFOR binds, and consequently the viability of the *rep uvrD* mutant becomes independent of the *recF* context in these RNA Pol mutants.

## Experimental procedures

### Strains and plasmids

The strain backgrounds are MG1655, or JJC40, which is an *hsdR* Thr^+^ Pro^+^ derivative of AB1157 (*leu-6 thi-1*, *his-4*, *argE3*, *lacY1*, *galK2*, *ara-14*, *xyl-5*, *mtl-1*, *tsx-33*, *rpsL31*, *supE44, hsdR, hsdM*). JJC147 is HfrPK3-PO131 (*thr1, leuB6, azi-15, tonA1, lacY1, supE44*, *argE86*::Tn*10*). Strains genotypes and plasmids are described in Table S1. InvA is described in Boubakri *et al*. (2010), it is an MG1655 derivative, deleted for *lacZ* and *att*λ, which carries a 18 kb inversion (NT 4 025 300 to 4 043 723) encompassing the *rrnA* operon. Minimum medium is M9 supplied with thiamine 0.05%, CaCl_2_ 100 µM, MgSO_4_ 2 mM, glucose 0.4% (Miller, 1992). Casamino acids 0.2% were added for strain background other than MG1655. Most of the strains were constructed by P1 transduction (Miller, 1992). For Hfr conjugations, a mix of 10% donor cells/90% recipient cells was incubated for 15 min with low agitation at 37°C before plating on selective media. Null mutants were checked by polymerase chain reaction with external oligonucleotides that amplify a DNA fragment of different length for the wild-type and the interrupted alleles. Oligonucleotides used for checking mutations are shown in Table S4. *uvrD* mutants were checked for their UV-sensitive phenotypes and for their mutator phenotype (about 100-fold increase in the proportion of Rif^R^ clones in overnight cultures). The plasmids pGBts-rep (Petit and Ehrlich, 2002), pAM-rep (Lestini and Michel, 2008) and pAM-priA (Grompone *et al*., 2004) were described; they carry the *rep* or the *priA* gene under the control of their own promoter; they were segregated as published.

*rpo* mutations are *rpoB* A1331G (*rpoB*^D444G^), A1340G (*rpoB*^H447R^), A1552G (*rpoB*^N518D^), *rpoC*A338G (*rpoC*^H113R^), C1352T (rpoC^P451L^). Following P1 transduction or conjugation, screening for A1552G (*rpoB*^N518D^) was based the Rif^R^ phenotype it confers. Screening for A1331G (*rpoB*^D444G^) was done sequencing the *rpoB* region. Three mutations were screened after polymerase chain reaction amplification of the region either by sequencing or by looking for the presence of a restriction site that they create: A1340G (*rpoB*^H447R^) creates a BsiEI site, *rpoC*A338G (*rpoC*^H113R^) creates a BssHII site, C1352T (rpoC^P451L^) creates a BspMI site. Upon P1 co-transduction of *rpo^sup^* with *thiC*::Tn*10* in a context where the mutation confers a phenotype, the phenotype and the presence of the mutation were tested in four to eight different Tet^R^ transductants and there was 100% correlation between the presence of the mutation and the appearance of the new phenotype. Twenty regularly spaced oligonucleotides in *rpoB* (10 on each strand) and 20 regularly spaced oligonucleotides in *rpoC* (10 on each strand) were used for sequencing of the entire genes.

### Measurement of plating efficiency

Overnight cultures grown in MM (OD_650_ 1 to 2) were diluted and plated on MM or LB plates, incubated at 37°C, 30°C or 25°C. LB plates at 37°C were counted after 24 and 48 h of incubation, MM plates at 37°C and LB plates at 30°C after 48 h and 3 days of incubation, and MM plates at 30°C and LB plates at 25°C after 3 and 4 days incubation.

### Measurement of UV sensitivity and of β-galactosidase activity

Survival to UV irradiation was performed as described (Baharoglu *et al*., 2006). β-galactosidase assays for the measure of P*_rrnB_* activity were performed as described (Miller, 1992).

## References

[b1] Aguilera A, Gomez-Gonzalez B (2008). Genome instability: a mechanistic view of its causes and consequences. Nat Rev Genet.

[b2] Azvolinsky A, Giresi PG, Lieb JD, Zakian VA (2009). Highly transcribed RNA polymerase II genes are impediments to replication fork progression in Saccharomyces cerevisiae. Mol Cell.

[b3] Baharoglu Z, Petranovic M, Flores MJ, Michel B (2006). RuvAB is essential for replication forks reversal in certain replication mutants. EMBO J.

[b4] Barker MM, Gaal T, Gourse RL (2001a). Mechanism of regulation of transcription initiation by ppGpp. II. Models for positive control based on properties of RNAP mutants and competition for RNAP. J Mol Biol.

[b5] Barker MM, Gaal T, Josaitis CA, Gourse RL (2001b). Mechanism of regulation of transcription initiation by ppGpp. I. Effects of ppGpp on transcription initiation in vivo and in vitro. J Mol Biol.

[b6] Bartlett MS, Gaal T, Ross W, Gourse RL (1998). RNA polymerase mutants that destabilize RNA polymerase-promoter complexes alter NTP-sensing by rrn P1 promoters. J Mol Biol.

[b7] Bartlett MS, Gaal T, Ross W, Gourse RL (2000). Regulation of rRNA transcription is remarkably robust: FIS compensates for altered nucleoside triphosphate sensing by mutant RNA polymerases at Escherichia coli rrn P1 promoters. J Bacteriol.

[b8] Borukhov S, Lee J, Laptenko O (2005). Bacterial transcription elongation factors: new insights into molecular mechanism of action. Mol Microbiol.

[b9] Boubakri H, de Septenville AL, Viguera E, Michel B (2010). The helicases DinG, Rep and UvrD cooperate to promote replication across transcription units in vivo. EMBO J.

[b10] Broccoli S, Rallu F, Sanscartier P, Cerritelli SM, Crouch RJ, Drolet M (2004). Effects of RNA polymerase modifications on transcription-induced negative supercoiling and associated R-loop formation. Mol Microbiol.

[b11] Garibyan L, Huang T, Kim M, Wolff E, Nguyen A, Nguyen T (2003). Use of the rpoB gene to determine the specificity of base substitution mutations on the Escherichia coli chromosome. DNA Repair (Amst).

[b12] Grompone G, Bidnenko V, Ehrlich SD, Michel B (2004). PriA is essential for viability of the Escherichia coli topoisomerase IV parE10(Ts) mutant. J Bacteriol.

[b13] Guy CP, Atkinson J, Gupta MK, Mahdi AA, Gwynn EJ, Rudolph CJ (2009). Rep provides a second motor at the replisome to promote duplication of protein-bound DNA. Mol Cell.

[b14] Korzheva N, Mustaev A, Kozlov M, Malhotra A, Nikiforov V, Goldfarb A, Darst SA (2000). A structural model of transcription elongation. Science.

[b15] Kuzminov A (1999). Recombinational repair of DNA damage in Escherichia coli and bacteriophage lambda. Microbiol Mol Biol Rev.

[b16] Lane HE, Denhardt DT (1975). The rep mutation. IV. Slower movement of replication forks in Escherichia coli rep strains. J Mol Biol.

[b17] Lestini R, Michel B (2007). UvrD controls the access of recombination proteins to blocked replication forks. EMBO J.

[b18] Lestini R, Michel B (2008). UvrD and UvrD252 counteract RecQ, RecJ, and RecFOR in a rep mutant of Escherichia coli. J Bacteriol.

[b19] Michel B, Boubakri H, Baharoglu Z, Lemasson M, Lestini R (2007). Recombination proteins and rescue of arrested replication forks. DNA Repair (Amst).

[b20] Miller JH (1992). A Short Course in Bacterial Genetic: Cold Spring Harbor.

[b21] Mirkin EV, Mirkin SM (2007). Replication fork stalling at natural impediments. Microbiol Mol Biol Rev.

[b22] Nudler E (2009). RNA polymerase active center: the molecular engine of transcription. Annu Rev Biochem.

[b23] Paul BJ, Berkmen MB, Gourse RL (2005). DksA potentiates direct activation of amino acid promoters by ppGpp. Proc Natl Acad Sci USA.

[b24] Petit MA, Ehrlich D (2002). Essential bacterial helicases that counteract the toxicity of recombination proteins. EMBO J.

[b25] Pomerantz RT, O'Donnell M (2010). Direct restart of a replication fork stalled by a head-on RNA polymerase. Science.

[b26] Potrykus K, Cashel M (2008). (p)ppGpp: still magical?. Annu Rev Microbiol.

[b27] Rudolph CJ, Dhillon P, Moore T, Lloyd RG (2007). Avoiding and resolving conflicts between DNA replication and transcription. DNA Repair (Amst).

[b28] Rutherford ST, Villers CL, Lee JH, Ross W, Gourse RL (2009). Allosteric control of Escherichia coli rRNA promoter complexes by DksA. Genes Dev.

[b29] Seigneur M, Bidnenko V, Ehrlich SD, Michel B (1998). RuvAB acts at arrested replication forks. Cell.

[b30] Szalewska-Palasz A, Johansson LU, Bernardo LM, Skarfstad E, Stec E, Brannstrom K, Shingler V (2007). Properties of RNA polymerase bypass mutants: implications for the role of ppGpp and its co-factor DksA in controlling transcription dependent on sigma54. J Biol Chem.

[b31] Taucher-Scholtz G, Abdel-Monem M, Hoffmann-Berling H, Cozzarelli NR (1983). Functions of helicases in *E. coli*. Mechanisms of DNA Replication and Recombination.

[b32] Trautinger BW, Lloyd RG (2002). Modulation of DNA repair by mutations flanking the DNA channel through RNA polymerase. EMBO J.

[b33] Trautinger BW, Jaktaji RP, Rusakova E, Lloyd RG (2005). RNA polymerase modulators and DNA repair activities resolve conflicts between DNA replication and transcription. Mol Cell.

[b34] Vassylyev DG, Sekine S, Laptenko O, Lee J, Vassylyeva MN, Borukhov S, Yokoyama S (2002). Crystal structure of a bacterial RNA polymerase holoenzyme at 2.6 A resolution. Nature.

[b35] Vassylyev DG, Vassylyeva MN, Perederina A, Tahirov TH, Artsimovitch I (2007a). Structural basis for transcription elongation by bacterial RNA polymerase. Nature.

[b36] Vassylyev DG, Vassylyeva MN, Zhang J, Palangat M, Artsimovitch I, Landick R (2007b). Structural basis for substrate loading in bacterial RNA polymerase. Nature.

[b37] Veaute X, Delmas S, Selva M, Jeusset J, Le Cam E, Matic I (2005). UvrD helicase, unlike Rep helicase, dismantles RecA nucleoprotein filaments in Escherichia coli. EMBO J.

[b38] Yancey-Wrona JE, Matson SW (1992). Bound Lac repressor protein differentially inhibits the unwinding reactions catalyzed by DNA helicases. Nucleic Acids Res.

[b39] Zhang G, Campbell EA, Minakhin L, Richter C, Severinov K, Darst SA (1999). Crystal structure of Thermus aquaticus core RNA polymerase at 3.3 A resolution. Cell.

[b40] Zhou YN, Jin DJ (1998). The rpoB mutants destabilizing initiation complexes at stringently controlled promoters behave like ‘stringent’ RNA polymerases in Escherichia coli. Proc Natl Acad Sci USA.

