# Supplementary material for: RNA polymerase mutations that facilitate replication progression in the rep uvrD recF mutant lacking two accessory replicative helicases
Source: Mol Microbiol. 2010 Jul;77(2):324–36. doi: 10.1111/j.1365-2958.2010.07208.x (PMC2936116; doi:10.1111/j.1365-2958.2010.07208.x)
Supplement: Supplementary file 1 [file mmi0077-0324-SD1.pdf]

Table S1 Strains and plasmids

Strain	Relevant genotype	Construction or reference
CAG18499	*zid* ::Tn*10*	(Nichols *et al.*, 1998)
CAG18500	*thiC* ::Tn*10*	(Nichols *et al.*, 1998)
JJC40	wild-type	Laboratory collection
JJC147	Hfr PK3-PO131 *argE86::Tn10*	CGSC6763 Genetic Stock Center
JJC212	*uvrD*::Tn*5*	Laboratory collection
JJC451	*recF400::*Tn*5*	Laboratory collection
JJC754	Δ*ruvABC::cm*	(Seigneur *et al.*, 1998)
JJC760	Δ*rep::cam* [pGBts-rep+]	(Petit and Ehrlich, 2002)
JJC213	Δ*rep::kan*	(Uzest *et al.*, 1991)
JJC1086	Δ*recB::kan* [pDWS2]	Δ*recB::kan* (Murphy, 1998) pDWS2: pBR322-RecBCD^{+} (G. Smith)
JJC1177	*rep::Ap*	Laboratory collection
JJC1519	Hfr PK3-PO131 *argE86::Tn10 recF400::*Tn*5*	JJC147 * P1 JJC450
JJC1706	Δ*rep::cam uvrD::*Tn*5 recO::*Tn*5 metE::*Tn*10* [pGBts-rep+]	MAC569 in (Petit and Ehrlich, 2002)
JJC1707	Δ*rep::cam uvrD::*Tn*5 recR::*Tn*5 metE::*Tn*10* [pGBts-rep+]	MAC 574 in (Petit and Ehrlich, 2002)
JJC1708	Δ*rep::cam uvrD::*Tn*5* [pGBts-rep+]	MAC 556 in (Petit and Ehrlich, 2002)
JJC1857	Δ*dinG::kan*	Gene replacement of *dinG* by Kan^{R} of pKD4 (Datsenko and Wanner, 2000)
JJC1861	Δ*dinG::cm*	(Boubakri et al, 2010)
JJC1892	*rep::Ap* [pAM-rep+]	Laboratory collection
JJC1945	*sfiA11*	(Grompone *et al.*, 2004)
JJC1960	*uvrD::*Tn*5 rep::Ap* [pAM-rep+]	Laboratory collection
JJC2062	*recF400::*Tn*5 zid501::*Tn*10*	Laboratory collection
JJC2177	Hfr PK3-PO131 *recF400::*Tn*5*	JJC1519* P1 JJC40
JJC2230	Δ*ruvABC::cm* [pGB-ruvABC+]	(Lestini and Michel, 2008)
JJC2386	Δ*(uvrD-yigB)::cam*	(Flores *et al.*, 2005)
JJC2418	*rep::Ap uvrD::*Tn*5* [pGBts-rep+]	JJC1708 P1 JJC1177
JJC2451	*rep::Ap uvrD::*Tn*5 recF400::*Tn*5* [pGBts-rep+]	JJC2418 * PA JJC451
JJC2488	*sfiA11 rep::Ap* Δ*(uvrD-yigB)::cam recO*::Tn*5* [pGBts-rep+]	Laboratory collection
JJC2634	Δ*uvrD294::Kan*	(Lestini and Michel, 2007)
JJC3122	Δ*rep::cam uvrD::*Tn*5 recQ1803*::Tn*3* [pGBts-rep+]	(Lestini and Michel, 2008)
JJC3870	Δ*rep::kan* [pAM-rep+]	JJC213 transformed with [pAM-rep+]
JJC3971	Δ*rep::kan* Δ*uvrD294::kan* [pAM-rep+]	JJC3870 * P1 JJC2634
JJC3998	Hfr PK3-PO131 *recF400::*Tn*5 rep::Ap*	JJC2177 * P1 JJC1177
JJC4013	Hfr PK3-PO131 *recF400::*Tn*5 rep::Ap* [pAM-rep]	JJC3998 transformed with pAM-rep
JJC4035	Hfr PK3-PO131 *recF400::*Tn*5 rep::Ap* Δ*(uvrD-yigB)::cam* [pAM-rep]	JJC4013 * P1 JJC2386
JJC4038	*rep::Ap uvrD::*Tn*5 recF400::*Tn*5* *rpoC*^{P451L} (S1)	LB- resistant derivative of JJC4048
JJC4039	*rep::Ap uvrD::*Tn*5 recF400::*Tn*5* *rpoC*^{H113R} (S2)	LB- resistant derivative of JJC4048
JJC4040	*rep::Ap uvrD::*Tn*5 recF400::*Tn*5* *rpoB*^{H447R} (S3m)	LB- resistant derivative of JJC4048
JJC4041	*rep::Ap uvrD::*Tn*5 recF400::*Tn*5* *rpoB*^{D444G} (S3a)	LB- resistant derivative of JJC4048
JJC4043	*rep::Ap uvrD::*Tn*5 recF400::*Tn*5* *rpoC*^{H113R} [pAM-rep] Δ*dinG::cm*	JJC4039 * pAM-rep * P1 JJC1861
JJC4044	*rep::Ap uvrD::*Tn*5 recF400::*Tn*5* *rpoB*^{H447R} [pAM-rep] Δ*dinG::cm*	JJC4040 * pAM-rep * P1 JJC1861

JJC4045	*rep::Ap uvrD::*Tn*5 recF400::*Tn*5 rpoB*^{D444G} [pAM-rep] Δ*dinG::cm*	JJC4041 * pAM-rep * P1 JJC1861
JJC4047	*rep::Ap uvrD::*Tn*5 recF400::*Tn*5 rpoC*^{P451L} [pAM-rep] Δ*dinG::cm*	JJC4038 * pAM-rep * P1 JJC1861
JJC4048	*rep::Ap uvrD::*Tn*5 recF400::*Tn*5*	JJC2451 cured of pBGts-rep
JJC4053	*rep::Ap uvrD::*Tn*5 recF400::*Tn*5 rpoC*^{H113R} (S3b)	LB- resistant derivative of JJC4048
JJC4100	Hfr PK3-PO131 *recF400::*Tn*5 rep::Ap* Δ*(uvrD-yigB)::cam rpoB*^{N518D}	Fast growing segregant of JJC4035
JJC4107	*relA*::*kan*	RLG3373 (Dr. R. Gourse)
JJC4108	*relA*::*kan spoT::cam*	RLG3373 (Dr. R. Gourse)
JJC4109	*rpoC*$^{\Delta 215\text{-}220}$ *thiC*::Tn*10*	RLG3381 in (Bartlett *et al.*, 1998)
JJC4112	*lacZ lacI lambda::P1*$_{-61+1\ rrnB}$ *lacZ*	RLG5950 in (Bartlett *et al.*, 1998)
JJC4113	*lacZ lacI lambda::P1*$_{-61+1\ rrnB}$ *lacZ greA::cam*	RLG7239 (Dr. R. Gourse)
JJC4114	*lacZ lacI lambda::P1*$_{-61+1\ rrnB}$ *lacZ greB::kan*	RLG7240 (Dr. R. Gourse)
JJC4124	*rep::Ap* Δ*(uvrD-yigB)::cam recF400::*Tn*5 rpoC*^{P451L}	JJC4038 * Hfr4100 selection for Cm^{R} Tet^{R} , screening for Rif^{S}
JJC4125	*rep::Ap* Δ*(uvrD-yigB)::cam recF400::*Tn*5 rpoB*^{H447R}	JJC4040 * Hfr4100 selection for Cm^{R} Tet^{R} , screening for Rif^{S}
JJC4126	*rep::Ap* Δ*(uvrD-yigB)::cam recF400::*Tn*5 rpoB*^{D444G}	JJC4041 * Hfr4100 selection for Cm^{R} Tet^{R} , screening for Rif^{S}
JJC4141	*rep::Ap* Δ*(uvrD-yigB)::cam recF400::*Tn*5 rpoC*^{H113R}	JJC4039 * Hfr4100 selection for Cm^{R} Tet^{R} , screening for Rif^{S}
JJC4142	*rep::Ap* Δ*(uvrD-yigB)::cam recF400::*Tn*5 rpoC*^{H113R}	JJC4053 * Hfr4100 selection for Cm^{R} Tet^{R} , screening for Rif^{S}
JJC4162	*rep::Ap* Δ*(uvrD-yigB)::cam recF400::*Tn*5 rpoC*^{P451L} *thiC*::Tn*10*	JJC4124 * P1 CGA18500
JJC4163	*rep::Ap* Δ*(uvrD-yigB)::cam recF400::*Tn*5 rpoB*^{H447R} *thiC*::Tn*10*	JJC4125 * P1 CAG18500
JJC4164	*rep::Ap* Δ*(uvrD-yigB)::cam recF400::*Tn*5 rpoB*^{D444G} *thiC*::Tn*10*	JJC4126 * P1 CAG18500
JJC4170	*rep::Ap* Δ*(uvrD-yigB)::cam recF400::*Tn*5 rpoC*^{H113R} *thiC*::Tn*10*	JJC4141 * P1 CAG18500
JJC4174	*sfiA11* Δ*recQ6215::Cm*	(Lestini and Michel, 2007)
JJC4186	*recF400::*Tn*5 rep::Ap* Δ*(uvrD-yigB)::cam rpoB*^{N518D}	JJC4048 * Hfr4100 selection for Cm^{R} Tet^{R} , screening for Rif^{R}
JJC4200	*recF400::*Tn*5 rep::Ap* Δ*(uvrD-yigB)::cam rpoB*^{N518D} *thiC*::Tn*10*	JJC4186 * P1 CAG18500
JJC4215	Δ*rep::kan* Δ*uvrD294::kan* [pAM-rep+] *rpoC*$^{\Delta 215\text{-}220}$ *thiC*::Tn*10*	JJC3971 * JJC4109
JJC4247	*rep::Ap* Δ*(uvrD-yigB)::cam zid*::Tn*10 rpoC*^{P451L} [pAM-rep]	JJC4124 * P1 CAG18999
JJC4248	*rep::Ap* Δ*(uvrD-yigB)::cam zid*::Tn*10 rpoB*^{H447R} [pAM-rep]	JJC4125* P1 CAG18999
JJC4249	*rep::Ap* Δ*(uvrD-yigB)::cam zid*::Tn*10 rpoB*^{D444G} [pAM-rep]	JJC4126*P1 CAG18999
JJC4251	*rep::Ap* Δ*(uvrD-yigB)::cam zid*::Tn*10 rpoC*^{H113R} [pAM-rep]	JJC4442 * P1 CAG18999
JJC4252	Hfr PK3-PO131 *zid*::Tn*10 rep::Ap* Δ*(uvrD-yigB)::cam rpoB*^{N518D} [pAM-rep]	JJC4100 * P1 CAG18999
JJC4257	*sfiA11 rep::Ap* Δ*(uvrD-yigB)::cam recO*::Tn*5 rpoB*^{N518D} *thiC*::Tn*10*	JJC2488 * P1 JJC4200 cured of pGBts-rep
JJC4281	Δ*rep::kan* Δ*uvrD294::kan* [pAM-rep+] *rpoB*^{H447R} *thiC*::Tn*10*	JJC3971 * P1 JJC4163
JJC4283	*sfiA11 rep::Ap* Δ*(uvrD-yigB)::cam recO*::Tn*5 rpoC*^{P451L} *thiC*::Tn*10*	JJC2488 * P1 JJC4162 cured of pGBts-rep
JJC4304	Δ*rep::kan* Δ*uvrD294::kan* [pAM-rep+] *rpoB*^{D444G} *thiC*::Tn*10*	JJC3971 * P1 JJC4164
JJC4324	*sfiA11 rep::Ap* Δ*(uvrD-yigB)::cam recO*::Tn*5 rpoB*^{D444G} *thiC*::Tn*10*	JJC2488 * P1 JJC4164 cured of pGBts-rep
JJC4340	*rep::Ap* Δ*(uvrD-yigB)::cam recF400::*Tn*5 rpoC*^{H113R} *thiC*::Tn*10*	JJC4142 * P1 CAG18500
JJC4342	*sfiA11 rep::Ap* Δ*(uvrD-yigB)::cam recO*::Tn*5 rpoB*^{H447R} *thiC*::Tn*10*	JJC2488 * P1 JJC4163 cured of pGBts-rep
JJC4344	*sfiA11 rep::Ap* Δ*(uvrD-yigB)::cam recO*::Tn*5 rpoC*^{H113R} *thiC*::Tn*10*	JJC2488 * P1 JJC4170 cured of pGBts-rep
JJC4354	Δ*rep::kan* Δ*uvrD294::kan* [pAM-rep+] *rpoC*^{P451L} *thiC*::Tn*10*	JJC3971 * P1 JJC4162

JJC4355	Δ*rep::kan* Δ*uvrD294::kan* [pAM-rep+] *rpoC*^{H113R} *thiC*::Tn*10*	JJC3971 * P1 JJC4170
JJC4488	*rpoB*^{N518D} *thiC*::Tn*10*	JJC40 * P1 JJC4200
JJC4489	*rpoC*^{P451L} *thiC*::Tn*10*	JJC40 * P1 JJC4162
JJC4490	*rpoB*^{D444G} *thiC*::Tn*10*	JJC40 * P1 JJC4164
JJC4491	*rpoB*^{H447R} *thiC*::Tn*10*	JJC40 * P1 JJC4163
JJC4512	*lacZ lacI lambda::P1*$_{-61+1\ rrnB}$ *lacZ rpoC*^{P451L} *thiC*::Tn*10*	JJC4112 * P1 JJC4162
JJC4514	*lacZ lacI lambda::P1*$_{-61+1\ rrnB}$ *lacZ rpoB*^{H447R} *thiC*::Tn*10*	JJC4112 * P1 JJC4163
JJC4515	*lacZ lacI lambda::P1*$_{-61+1\ rrnB}$ *lacZ rpoC*^{N518D} *thiC*::Tn*10*	JJC4112 * P1 JJC4200
JJC4525	*lacZ lacI lambda::P1*$_{-61+1\ rrnB}$ *lacZ rpoC*^{H113R} *thiC*::Tn*10*	JJC4112 * P1 JJC4170
JJC4528	*rpoC*$^{\Delta 215-220}$ *thiC*::Tn*10*	JJC40 * P1 JJC4109
JJC4531	*rpoC*^{H113R} *thiC*::Tn*10*	JJC40 * P1 JJC4340
JJC4532	*rpoC*^{H113R} *thiC*::Tn*10*	JJC40 *P1 JJC4170
JJC4547	*rpoC*^{N518D} *thiC*::Tn*10* Δ*ruvABC::cm*	JJC4488 * P1 JJC2230
JJC4548	*rpoC*^{P451L} *thiC*::Tn*10* Δ*ruvABC::cm*	JJC4488 * P1 JJC2230
JJC4549	*rpoB*^{D444G} *thiC*::Tn*10* Δ*ruvABC::cm*	JJC4490 * P1 JJC2230
JJC4553	*rpoC*^{N518D} *thiC*::Tn*10 relA::kan*	JJC4488 * P1 JJC4107
JJC4554	*rpoC*^{P451L} *thiC*::Tn*10 relA::kan*	JJC4489 * P1 JJC4107
JJC4555	*rpoB*^{D444G} *thiC*::Tn*10 relA::kan*	JJC4490 * P1 JJC4107
JJC4556	*rpoB*^{H447R} *thiC*::Tn*10 relA::kan*	JJC4491 * P1 JJC4107
JJC4557	*rpoC*^{H113R} *thiC*::Tn*10 relA::kan*	JJC4531 * P1 JJC4107
JJC4558	*rpoC*^{H113R} *thiC*::Tn*10 relA::kan*	JJC4532 * P1 JJC4107
JJC4719	*rep::cam uvrD*::Tn*5 rpoC*$^{\Delta 215-220}$ *thiC*::Tn*10* [pGBts-rep+]	JJC1708 * P1 JJC4528
JJC4818	*rpoB*^{N518D} *thiC*::Tn*10 greB::kan greA::cam*	[JJC4488 *P14205] * P1 4204
JJC4820	*rpoB*^{D444G} *thiC*::Tn*10 greB::kan greA::cam*	[JJC4490 *P14205] * P1 4204
JJC4821	*rpoB*^{H447R} *thiC*::Tn*10 greB::kan greA::cam*	[JJC4491 *P14205] * P1 4204
JJC4822	*rpoC*^{H113R} *thiC*::Tn*10 greB::kan greA::cam*	[JJC4531 * P14205] * P1 4204
JJC4823	*rpoC*^{H113R} *thiC*::Tn*10 greB::kan greA::cam*	[JJC4532 *P14205] * P1 4204
JJC4824	*greB::kan greA::cam*	[JJC40 *P14205] * P1 4204
JJC4828	InvA Δ*dinG* Δ*rep* ::cm^{R} [pAM-rep]	(Boubakri *et al.*, 2010)
JJC4832	*rpoB*^{H447R} *thiC*::Tn*10* Δ*ruvABC::cm*	JJC4491 * P1 JJC2230
JJC4860	*rpoB*^{N518D} *thiC*::Tn*10* Δ*recQ::cam*	JJC4488 *P1 JJC4174
JJC4861	*rpoC*^{P451L} *thiC*::Tn*10* Δ*recQ::cam*	JJC4489 *P1 JJC4174
JJC4862	*rpoB*^{D444G} *thiC*::Tn*10* Δ*recQ::cam*	JJC4490 *P1 JJC4174
JJC4863	*rpoB*^{H447R} *thiC*::Tn*10* Δ*recQ::cam*	JJC4491 *P1 JJC4174
JJC4864	*rpoC*$^{\Delta 215-220}$ *thiC*::Tn*10* Δ*recQ::cam*	JJC4528 *P1 JJC4174
JJC4865	*rpoC*^{H113R} *thiC*::Tn*10* Δ*recQ::cam*	JJC4532 *P1 JJC4174
JJC4986	*rpoC*$^{\Delta 215-220}$ *thiC*::Tn*10* Δ*ruvABC::cm*	JJC4528 * P1 JJC2230
JJC4895	*rpoB*^{N518D} *thiC*::Tn*10 rep::Ap uvrD::*Tn*5* [pGBts-rep+]	JJC4488 [pGBts-rep]* P1 JJC2418
JJC4896	*rpoB*^{D444G} *thiC*::Tn*10 rep::Ap uvrD::*Tn*5* [pGBts-rep+]	JJC4490 [pGBts-rep]* P1 JJC2418
JJC4897	*rpoC*$^{\Delta 215-220}$ *thiC*::Tn*10 rep::Ap uvrD::*Tn*5* [pGBts-rep+]	JJC4528 [pGBts-rep]* P1 JJC2418
JJC4898	*rpoC*^{H113R} *thiC*::Tn*10 rep::Ap uvrD::*Tn*5* [pGBts-rep+]	JJC4532 [pGBts-rep]* P1 JJC2418
JJC4899	*rpoC*^{P451L} *thiC*::Tn*10 rep::Ap uvrD::*Tn*5* [pGBts-rep+]	JJC4489 [pGBts-rep]* P1 JJC2418
JJC4900	*rpoB*^{H447R} *thiC*::Tn*10 rep::Ap uvrD::*Tn*5* [pGBts-rep+]	JJC4491 [pGBts-rep]* P1 JJC2418
JJC4901	*rpoB*^{N518D} *thiC*::Tn*10* Δ*recQ::cam rep::Ap uvrD::*Tn*5* [pGBts-rep+]	JJC4860 [pGBts-rep] * P1 JJC2418

JJC4902	*rpoC*^{P451L} *thiC*::Tn*10* Δ*recQ::cam rep::Ap uvrD::*Tn*5* [pGBts-rep+]	JJC4861 [pGBts-rep] * P1 JJC2418
JJC4903	*rpoB*^{D444G} *thiC*::Tn*10* Δ*recQ::cam rep::Ap uvrD::*Tn*5* [pGBts-rep+]	JJC4862 [pGBts-rep] * P1 JJC2418
JJC4904	*rpoB*^{H447R} *thiC*::Tn*10* Δ*recQ::cam rep::Ap uvrD::*Tn*5* [pGBts-rep+]	JJC4863 [pGBts-rep] * P1 JJC2418
JJC4905	*rpoC*$^{\Delta 215\text{-}220}$ *thiC*::Tn*10* Δ*recQ::cam rep::Ap uvrD::*Tn*5* [pGBts-rep+]	JJC4864 [pGBts-rep] * P1 JJC2418
JJC4906	*rpoC*^{H113R} *thiC*::Tn*10* Δ*recQ::cam rep::Ap uvrD::*Tn*5* [pGBts-rep+]	JJC4865 [pGBts-rep] * P1 JJC2418
JJC4911	*rpoB*^{D444G} *thiC*::Tn*10 rep::Ap uvrD::*Tn*5* [pGBts-rep+] Δ*dinG::cm*	JJC4896 * P1 JJC1861
JJC4912	*rpoC*$^{\Delta 215\text{-}220}$ *thiC*::Tn*10 rep::Ap uvrD::*Tn*5* [pGBts-rep+] Δ*dinG::cm*	JJC4897 * P1 JJC1861
JJC4913	*rpoC*^{H113R} *thiC*::Tn*10 rep::Ap uvrD::*Tn*5* [pGBts-rep+] Δ*dinG::cm*	JJC4898 * P1 JJC1861
JJC4918	*rpoB*^{H447R} *thiC*::Tn*10 rep::Ap uvrD::*Tn*5* [pGBts-rep+] Δ*dinG::cm*	JJC4900 * P1 JJC1861
JJC4940	*InvA* Δ*dinG*	(Boubakri *et al.*, 2010)
JJC4962	*InvA* Δ*dinG rpoC*$^{\Delta 215\text{-}220}$ *thiC*::Tn*10*	(Boubakri *et al.*, 2010)
JJC5136	Δ*rep* ::kan^{R} Δ*uvrD294* ::kan^{R} *recF400* ::Tn*5* [pAM-rep]	(Boubakri *et al.*, 2010)
JJC5151	Δ*rep* ::kanR Δ*uvrD294* ::kan^{R} *recF400* ::Tn*5* Δ*dinG* ::Cm^{R} [pAM-rep]	(Boubakri *et al.*, 2010)
JJC5152	Δ*rep* ::kanR Δ*uvrD294* ::kan^{R} *recF400* ::Tn*5* *rpoC*$^{\Delta 215\text{-}220}$ *thiC*::Tn10 [pAM-rep]	(Boubakri *et al.*, 2010)
JJC5153	Δ*rep* ::kanR Δ*uvrD294* ::kan^{R} *recF400* ::Tn*5* *rpoC*$^{\Delta 215\text{-}220}$ *thiC*::Tn10	(Boubakri *et al.*, 2010)
JJC5166	Δ*rep* ::kan^{R} Δ*uvrD294* ::kan^{R} *recF*::Tn5 *zid*::Tn10 [pAM-rep]	(Boubakri *et al.*, 2010)
JJC5169	Δ*rep* ::kan^{R} Δ*uvrD294* ::kan^{R} *recF400* ::Tn*5* Δ*dinG* ::Cm^{R} *rpoC*$^{\Delta 215\text{-}220}$ *thiC*::Tn10 [pAM-rep]	(Boubakri *et al.*, 2010)
JJC5170	Δ*rep* ::kan^{R} Δ*uvrD294* ::kan^{R} *recF400* ::Tn*5* *rpoC*$^{\Delta 215\text{-}220}$ *thiC*::Tn10 Δ*dinG* ::Cm^{R} [pAM-rep]	(Boubakri *et al.*, 2010)
JJC5236	*InvA* Δ*dinG rpoB*^{H447R} *thiC*::Tn*10*	JJC4940 * P1 JJC4163
JJC5237	*InvA* Δ*dinG rpoC*^{H113R} *thiC*::Tn*10*	JJC4940 * P1 JJC4170
JJC5238	*InvA* Δ*dinG rpoC*^{H113R} *thiC*::Tn*10*	JJC4940 * P1 JJC4340
JJC5239	*InvA* Δ*dinG rpoB*^{D444G} *thiC*::Tn*10*	JJC4940 * P1 JJC4164
JJC5241	*InvA* Δ*dinG rpoB*^{N518D} *thiC*::Tn*10*	JJC4940 * P1 JJC4200
JJC5253	*rep::Ap* Δ*(uvrD-yigB)::cam zid*::Tn*10* Δ*dinG::kan rpoC*^{P451L} [pAM-rep]	JJC4247 * P1 JJC1857
JJC5254	*rep::Ap* Δ*(uvrD-yigB)::cam zid*::Tn*10* Δ*dinG::kan rpoB*^{H447R} [pAM-rep]	JJC4248 * P1 JJC1857
JJC5255	*rep::Ap* Δ*(uvrD-yigB)::cam zid*::Tn*10* Δ*dinG::kan rpoB*^{D444G} [pAM-rep]	JJC4249 * P1 JJC1857
JJC5257	*rep::Ap* Δ*(uvrD-yigB)::cam zid*::Tn*10* Δ*dinG::kan rpoC*^{H113R} [pAM-rep]	JJC4251 * P1 JJC1857
JJC5258	Hfr PK3-PO131 *zid*::Tn*10 rep::Ap* Δ*(uvrD-yigB)::cam rpoB*^{N518D}	JJC4253 * P1 JJC1857
JJC5259	*rep::Ap uvrD::*Tn*5 recF400::*Tn*5* [pGBts-rep+] [pEM001]	JJC2451 transformed with pEM001
JJC5261	*sfiA11 rep::Ap* Δ*(uvrD-yigB)::cam recO*::Tn*5 rpoC*$^{\Delta 215\text{-}220}$ *thiC*::Tn10 [pGBts-rep+]	JJC2488 * P JJC4528
JJC5267	InvA Δ*dinG* Δ*rep* ::cm^{R} *rpoB*^{H447R} *thiC*::Tn*10* [pAM-rep]	JJC4828 * P1 JJC4163
JJC5268	InvA Δ*dinG* Δ*rep* ::cm^{R} *rpoC*^{H113R} *thiC*::Tn*10* [pAM-rep]	JJC4828 * P1 JJC4340
JJC5269	InvA Δ*dinG* Δ*rep* ::cm^{R} *rpoB*^{N518D} *thiC*::Tn*10* [pAM-rep]	JJC4828 * P1 JJC4200
JJC5308	InvA Δ*dinG* Δ*rep* ::cm^{R} *rpoB*^{D444G} *thiC*::Tn*10* [pAM-rep]	JJC4828 * P1 JJC4164
JJC5309	*rpoB*^{N518D} *thiC*::Tn*10 rep::Ap uvrD::*Tn*5* [pGBts-rep+] Δ*recQ::cam*	JJC4895 * P1 JJC4174
JJC5310	*rpoB*^{N518D} *thiC*::Tn*10 rep::Ap uvrD::*Tn*5* [pGBts-rep+] Δ*dinG::cm*	JJC4895 * P1 JJC1861
JJC5312	*rpoC*^{P451L} *thiC*::Tn*10 rep::Ap uvrD::*Tn*5* [pGBts-rep+] Δ*dinG::cm*	JJC4899 * P1 JJC1861
JJC5313	*rpoC*^{P451L} *thiC*::Tn*10 rep::Ap uvrD::*Tn*5* [pGBts-rep+] Δ*recQ::cam*	JJC4899 * P1 JJC4174
JJC5344	InvA Δ*dinG* Δ*rep* ::cm^{R} *rpoC*^{P451L} *thiC*::Tn*10* [pAM-rep]	JJC4828 * P1 JJC4162

JJC5351	*rep::Ap* Δ*(uvrD-yigB)::cam zid*::Tn*10 rpoC*^{H113R} [pAM-rep]	JJC4141 * P1 CAG18999
JJC5400	*InvA* Δ*dinG rpoC*^{P451L} *thiC*::Tn*10*	JJC4940 * P1 JJC4162
JJC5405	Δ*rep* ::kanR Δ*uvrD294* ::kan^{R} *recF400* ::Tn*5* Δ*dinG* ::Cm^{R} *rpoB*^{N518D} *thiC*::Tn*10* [pAM-rep]	JJC5151 * P1 JJC4200
JJC5406	Δ*rep* ::kanR Δ*uvrD294* ::kan^{R} *recF400* ::Tn*5* Δ*dinG* ::Cm^{R} *rpoC*^{P451L} *thiC*::Tn*10* [pAM-rep]	JJC5151 * P1 JJC4162
JJC5407	Δ*rep* ::kanR Δ*uvrD294* ::kan^{R} *recF400* ::Tn*5* Δ*dinG* ::Cm^{R} *rpoC*^{H113R} *thiC*::Tn*10* [pAM-rep]	JJC5151 * P1 JJC4340
JJC5425	Δ*rep* ::kanR Δ*uvrD294* ::kan^{R} *recF400* ::Tn*5* Δ*dinG* ::Cm^{R} *rpoB*^{D444G} *thiC*::Tn*10* [pAM-rep]	JJC5151 * P1 JJC4164
JJC5426	Δ*rep* ::kanR Δ*uvrD294* ::kan^{R} *recF400* ::Tn*5* Δ*dinG* ::Cm^{R} *rpoB*^{H447R} *thiC*::Tn*10* [pAM-rep]	JJC5151 * P1 JJC4163
JJC5449	*greB::kan*	JJC40 *P14205
JJC5450	*rpoC*^{P451L} *thiC*::Tn*10 greB::kan*	JJC4489 *P14205
JJC5455	*greB::kan greA::cam*	JJC5449* P1 4204
JJC5456	*rpoC*^{P451L} *thiC*::Tn*10 greB::kan greA::cam*	JJC5450 * P1 4204
JJC5469	*lacZ lacI lambda::P1*$_{-61+1\ rrnB}$ *lacZ rpoB*^{D444G} *thiC*::Tn*10*	JJC4112 * P1 JJC4164
JJC5523	*rpoC*^{N518D} *thiC*::Tn*10 relA::kan spoT::kan*	JJC4553 * P1 JJC4108
JJC5524	*rpoC*^{P451L} *thiC*::Tn*10 relA::kan spoT::kan*	JJC4554 * P1 JJC4108
JJC5525	*rpoB*^{D444G} *thiC*::Tn*10 relA::kan spoT::kan*	JJC4555 * P1 JJC4108
JJC5526	*rpoB*^{H447R} *thiC*::Tn*10 relA::kan spoT::kan*	JJC4556 * P1 JJC4108
JJC5527	*rpoC*^{H113R} *thiC*::Tn*10 relA::kan spoT::kan*	JJC4557 * P1 JJC4108
JJC5528	*rpoC*^{H113R} *thiC*::Tn*10 relA::kan spoT::kan*	JJC4558* P1 JJC4108
JJC5532	*rpoB*^{N518D} *thiC*::Tn*10* [pAM-rep] Δ*recB::kan*	JJC4488 [pAM-rep] * P1 JJC1086
JJC5533	*rpoC*^{P451L} *thiC*::Tn*10* [pAM-rep] Δ*recB::kan*	JJC4489 [pAM-rep] * P1 JJC1086
JJC5534	*rpoC*^{H113R} *thiC*::Tn*10* [pAM-rep] Δ*recB::kan*	JJC4532 [pAM-rep] * P1 JJC1086
JJC5535	*rpoB*^{D444G} *thiC*::Tn*10* [pAM-rep] Δ*recB::kan*	JJC4490 [pAM-rep] * P1 JJC1086
JJC5536	*rpoB*^{H447R} *thiC*::Tn*10* [pAM-rep] Δ*recB::kan*	JJC4491 [pAM-rep] * P1 JJC1086
JJC5540	*rpoC*$^{\Delta 215\text{-}220}$ *thiC*::Tn10 *priA2::kan* [pAM-priA]	MG1655 *priA2*::kan [pAM-priA] * P1 JJC4528
JJC5541	*rpoC*^{H113R} *thiC*::Tn10 *priA2::kan* [pAM-priA]	MG1655 *priA2*::kan [pAM-priA] * P1 JJC4170

Plasmids

Name	Description	Reference
pGBts-rep	pGB2ts vector carrying the *rep* gene under the control of its own promoter	(Petit and Ehrlich, 2002)
pAM-rep	pAM34 vector carrying the *rep* gene under the control of its own promoter	(Lestini and Michel, 2008)
pAM-priA	pAM34 vector carrying the *priA* gene under the control of its own promoter	(Grompone *et al.*, 2004)
pEM001	pACYC184 vector carrying the *rnh* (RnaseH) gene under the control of its own promoter	(Masse *et al.*, 1997)
pRL385	pUC vector carrying the *rpoB* wild-type gene under the control of Pr-*lac* (IPTG inducible) promoter	(Bartlett *et al.*, 1998))

Table S2 Complementation of the *rpo*^{sup} mutations with a pUC-RpoB^{+} plasmid

	MM 37°C p-RpoB+		LB 25°C pRpoB+	
	-IPTG	+IPTG	-IPTG	+IPTG
S3m- *rpoB*^{H447R}	$1.9\ 10^{9} \pm 1.5\ 10^{8}$	$1.6\ 10^{9} \pm 2.8\ 10^{8}$	$1.8\ 10^{9} \pm 2.5\ 10^{8}$	$<1\ 10^{7}$
S3a- *rpoB*^{D444G}	$9\ 10^{7} \pm 4.2\ 10^{7}$	$2.5\ 10^{8} \pm 6.3\ 10^{7}$	$4.4\ 10^{8} \pm 3\ 10^{8}$	$2\ 10^{8} \pm 1.3\ 10^{8}$
S1- *rpoC*^{P541L}	$7.5\ 10^{8} \pm 5.3\ 10^{7}$	$1.5\ 10^{9} \pm 7.6\ 10^{8}$	$1.4\ 10^{8} \pm 8.7\ 10^{7}$	$1.1\ 10^{9} \pm 1.5\ 10^{8}$
S3b- *rpoC*^{H113R}	$2.1\ 10^{9} \pm 4.2\ 10^{8}$	$1.9\ 10^{9} \pm 3.5\ 10^{8}$	$9.5\ 10^{8} \pm 3.5\ 10^{8}$	$1.7\ 10^{9} \pm 2.9\ 10^{8}$

The plasmid pRL385 (a pUC derived plasmid that carries the *rpoB* wild-type gene under the control of Pr-lac (IPTG inducible) {Bartlett, 1998 #11951}), was introduced by transformation in the four *rep uvrD recF rpo*^{mut} strains, selecting on MM ampicillin (Ap) at 37°C. Transformants were grown ON in MM Ap at 37°C and plated on MM Ap or LB Ap plates, containing (+IPTG) or not (– IPTG) IPTG. MM plates were incubated at 37°C and LB plates at 25°C. The number of colony forming units (cfu) is indicated here. Since the *rep uvrD recF* Rpo^{+} cells are cryo-sensitive on LB at 25°C, complementation of an *rpoB* mutation by the wild-type *rpoB* allele is expected to confer cryo-sensitivity.

Table S3 Out of the 5 different *rpo*^{sup} mutations, only *rpoB*^{H447R} suppresses the auxotrophy of a *relA spoT* mutant.

Strain	genotype	Δ*spoT*::Cm^{R} transductants		Name of the *rpoB*^{sup} *relA spoT* mutant$^{(b)}$ and growth on MM$^{(a)}$
		LB	MM$^{(a)}$	
JJC4553	Δ*relA*::Kan^{R} *rpoB*^{N518D}	146	0	JJC5523 -
JJC4554	Δ*relA*::Kan^{R} *rpoC*^{P541L}	148	0	JJC5524 -
JJC4555	Δ*relA*::Kan^{R} *rpoB*^{D444G}	230	0	JJC5525 -
JJC4556	Δ*relA*::Kan^{R} *rpoB*^{H447R}	166	160	JJC5526 +
JJC4557	Δ*relA*::Kan^{R} *rpoC*^{H113R}	172	0	JJC5527 -
JJC4558	Δ*relA*::Kan^{R} *rpoC*^{H113R}	100	0	JJC5528 -
JJC4559	Δ*relA*::Kan^{R} Rpo+	60	0	JJC5529 -

(a) Because the experiment is done in a JJC40 background, which requires histidine, leucine and arginine for growth, these three amino acids were included in the MM glucose plates.
(b) The *rpoB*^{sup} *relA spoT* mutants were propagated on LB and tested on MM glucose supplemented with histidine, leucine and arginine; they are also described in Table S1.

Table S4 Oligonucleotides used for checking mutations and sequencing.

Purpose (name)	Sequence (5’ to 3’)
delta-dinG::kan	TCTCTTATTTGATTCCCGGCATCGCCATTGCCCGCGAAGAGTGTGTAGGCTGGAGCTGCTTC
delta-dinG::kan	AACAAGTCCAGCTGTAGCCGATACCACGGCGCGGTGATTTCATATGAATATCCTCCTTA
To check dinG (dinG1)	ATGTTGGTGGTTATTGCGAGCC
To check dinG (dinGa)	GCGCAGTATCCCGGTCTAAATC
To check rep (ext1)	CTTACACGCGGTCACATTC
To check rep (int4)	CGGCCTTGATTATTTCCC
To check rpoC$^{\Delta 215\text{-}220}$ (ext1)	CTGGAAGACGAGTAATTCTCT
To check rpoC$^{\Delta 215\text{-}220}$ (rpoCi)	GTTTGATACGCTTGGTCAGC
To check recF (recF1)	CGTGTCGCCGATATACA
To check recF (recFa)	GACGTTACCTATAGCGG
To check priA (priA1)	ATCCGCACTCTTCTACGG
To check priA (priAa)	GCGTGAAAACGGTTACAG

References

Bartlett, M.S., Gaal, T., Ross, W., and Gourse, R.L. (1998) RNA polymerase mutants that destabilize RNA polymerase-promoter complexes alter NTP-sensing by rrn P1 promoters. *J Mol Biol* **279**: 331-345.

Boubakri, H., de Septenville, A.L., Viguera, E., and Michel, B. (2010) The helicases DinG, Rep and UvrD cooperate to promote replication across transcription units in vivo. *Embo J* **29**: 145-157.

Datsenko, K.A., and Wanner, B.L. (2000) One-step inactivation of chromosomal genes in Escherichia coli K-12 using PCR products. *Proc Natl Acad Sci U S A* **97**: 6640-6645.

Flores, M.J., Sanchez, N., and Michel, B. (2005) A fork-clearing role for UvrD. *Mol. Microbiol.* **57**: 1664-1675.

Grompone, G., Bidnenko, V., Ehrlich, S.D., and Michel, B. (2004) PriA is essential for viability of the Escherichia coli topoisomerase IV parE10(Ts) mutant. *J Bacteriol* **186**: 1197-1199.

Lestini, R., and Michel, B. (2007) UvrD controls the access of recombination proteins to blocked replication forks. *Embo J* **26**: 3804-3814.

Lestini, R., and Michel, B. (2008) UvrD and UvrD252 counteract RecQ, RecJ, and RecFOR in a rep mutant of Escherichia coli. *J Bacteriol* **190**: 5995-6001.

Masse, E., Phoenix, P., and Drolet, M. (1997) DNA topoisomerases regulate R-loop formation during transcription of the rrnB operon in Escherichia coli. *J Biol Chem* **272**: 12816-12823.

Murphy, K.C. (1998) Use of bacteriophage lambda recombination functions to promote gene replacement in Escherichia coli. *Journal of Bacteriology* **180**: 2063-2071.

Nichols, B.P., Shafiq, O., and Meiners, V. (1998) Sequence analysis of Tn10 insertion sites in a collection of Escherichia coli strains used for genetic mapping and strain construction. *J Bacteriol* **180**: 6408-6411.

Petit, M.A., and Ehrlich, D. (2002) Essential bacterial helicases that counteract the toxicity of recombination proteins. *Embo J* **21**: 3137-3147.

Seigneur, M., Bidnenko, V., Ehrlich, S.D., and Michel, B. (1998) RuvAB acts at arrested replication forks. *Cell* **95**: 419-430.

Uzest, M., Ehrlich, S.D., and Michel, B. (1991) The Escherichia coli terB sequence affects maintenance of a plasmid with the M13 phage replication origin. *J Bacteriol* **173**: 7695-7697.
